# Impact of IS*26* mobilization on genetic manipulation of multidrug-resistant *Acinetobacter baumannii*

**DOI:** 10.3389/fmicb.2025.1689239

**Published:** 2025-10-08

**Authors:** Yuan Hu, Junjie Zheng, Yanan Gong, Lihua He, Fanliang Meng, Jianzhong Zhang

**Affiliations:** ^1^State Key Laboratory of Infectious Disease Prevention and Control, Collaborative Innovation Center for Diagnosis and Treatment of Infectious Diseases, National Institute for Communicable Disease Control and Prevention, Chinese Center for Disease Control and Prevention, Beijing, China; ^2^The Fifth Medical Center of Chinese PLA General Hospital, Beijing, China

**Keywords:** *Acinetobacter baumannii*, insertion sequence, IS*26*, *pitA*, genetic manipulation

## Abstract

**Introduction:**

Multidrug-resistant *Acinetobacter baumannii* poses significant challenges for genetic manipulation, historically hindering research on this organism.

**Methods:**

To elucidate the factors contributing to these difficulties, *comA* and *xcpW* knockouts were performed on a multidrug-resistant (MDR) clinical isolate of international clone 2 (IC2), designated HN85.

**Results:**

Through electroporation, both constructed *telR*-marked suicide plasmids were recruited via active IS*26* transposition into adjacent genomic regions, complicating attempts to delete the target genes through homologous recombination. Transferred by natural transformation and conjugation methods, the suicide plasmids successfully evaded targeting by IS*26* and ultimately achieved *comA* and *xcpW* knockouts. During mutant screening following transformation, false positive colonies consistently emerged on tellurite plates without undergoing plasmid integration. Genomic sequencing revealed that this tellurite resistance resulted from the interruption of *pitA* caused by IS*26* transposition. To investigate whether the high transposition activity of IS*26* was attributable to its high copy number in HN85, a single IS*26* copy was introduced into a susceptible clinical *A. baumannii* isolate W068. Although W068 possesses a higher density of insertion sequence (IS) elements, IS*26* remained preferentially mobilized and exhibited similar active transposition behavior in the new host cell.

**Discussion:**

IS26 is prevalent in *A. baumannii* genomes (78.1%, 698/931), particularly among strains belonging to IC2 (99.8%, 509/510), implying its significant role in the evolution and success of IC2. The potential implications of active IS26 transposition for gene editing and screening warrant careful consideration beyond just *A. baumannii*.

## Introduction

*Acinetobacter baumannii* is a Gram-negative nosocomial pathogen implicated in various infections. It has garnered substantial attention due to escalating levels of antibiotic resistance and high mortality rates observed among critically ill patients. As one of the most concerning multidrug-resistant ESKAPE pathogens, *A. baumannii* poses significant challenges for treatment owing to its multiple intrinsic and acquired resistance mechanisms, which have contributed to the emergence of multidrug-resistant (MDR), extensively drug-resistant, and even pan-drug-resistant phenotypes (Peleg et al., 2008). Consequently, the World Health Organization (WHO) has classified carbapenem-resistant *A. baumannii* as part of “Priority 1: Critical group” for research (Tacconelli et al., 2018).

The population structure of *A. baumannii* isolates is quite diverse; however, only a limited number of specific clones, referred to as international clonal lineages (IC), are distributed globally. The widespread dissemination of carbapenem resistance can largely be attributed to the proliferation of two major clones known as international clone 1 (IC1) and international clone 2 (IC2). Although some new ICs have been identified, most are considered prevalent primarily within specific regions over certain periods (Al-Hassan et al., 2021; Muller et al., 2023; Shelenkov et al., 2023). IC2, designated by the Pasteur multi-locus sequence typing (MLST) scheme as ST2, remains the predominant and most widely distributed clone of *A. baumannii* (Hamidian and Nigro, 2019).

Numerous resistance mechanisms have been documented in *A. baumannii*; however, our current understanding of this organism is still limited. This limitation primarily arises from the fact that many contemporary gene editing tools and experimental protocols are developed and optimized using model strains, which may not accurately represent the problematic clonal lineages currently encountered in clinical settings. Consequently, unforeseen difficulties may emerge when these methods are applied to MDR clinical isolates.

For *A. baumannii*, conventional gene deletion strategies typically use a two-step method involving integration and excision. This process utilizes a specially constructed suicide knockout vector containing homologous regions along with selection and counter-selection markers for allelic exchange screening (Biswas, 2015). Antibiotic resistance genes are the most commonly utilized selectable markers; however, they are unsuitable for MDR strains. The tellurite resistance operon (*telR*), consisting of three consecutive genes—*kilA*, *telA*, and *telB*—has been reported to serve as an effective selection marker for knockout plasmids designed to target *A. baumannii* isolates, regardless of their antibiotic resistance profiles (Amin et al., 2013).

IS*26* plays a critical role in disseminating antibiotic resistance genes among Gram-negative bacteria (Partridge et al., 2018). It encodes the DDE transposase Tnp26, which possesses unique properties. Instead of moving independently to new locations, IS*26* transposes through a mechanism that exclusively forms cointegrates between two DNA molecules. This process results in a structure comprising a single copy of IS and an adjacent DNA segment known as the translocatable unit (TU), fused to a target molecule, thereby creating what is referred to as pseudo-compound transposon (PCT) structures via two directly-oriented copies of IS*26* (Harmer et al., 2020; Harmer and Hall, 2024). When the target site lacks IS*26*, the second IS copy is generated through a replicative step described as “copy in” mode, resulting in an 8 bp target site duplication (TSD) (Iida et al., 1984; Harmer and Hall, 2021). Conversely, when the target already contains IS*26*, Tnp26-mediated cointegrate formation can occur via a conservative route where neither the IS nor the target site undergoes duplication (Harmer et al., 2014). These cointegrates can subsequently be resolved through homologous recombination processes yielding two replicons—each carrying one copy of the IS (Harmer et al., 2020). Additionally, IS*26* can act on the same DNA molecule leading to either deletion or inversion of DNA located between itself and its target site.

In our study, we selected an MDR clinical isolate of IC2 *A. baumannii*, designated HN85, and conducted gene knockout experiments utilizing various transformation methods to clarify the factors hindering classical gene knockout technology. The involvement of IS*26* transposition was observed. Given that IS*26* is widely distributed among Gram-negative bacteria, comprehending the impact of its active transposition on gene editing is of significant importance.

## Materials and methods

### Bacterial strains, plasmids and culture conditions

Bacterial strains and plasmids used in this study are detailed in Table 1. *A. baumannii* strain HN85 and W068 were selected from a survey evaluating antimicrobial resistance and natural transformation capabilities among clinical isolates of *A. baumannii*. All strains are derivatives of wild-type clinical isolates *A. baumannii* HN85 or W068. Bacteria were cultured in lysogeny broth (LB). For selection purposes, growth media were supplemented with potassium tellurite (30 mg/L), kanamycin (50 mg/mL), or zeocin (250 μg/mL).

**Table 1:** Strains and plasmids used in this study.

Strain or plasmid	Relevant characteristics	Reference or source
Plasmid		
pRK2013	KmR, ColE1, tra+. Conjugative helper plasmid.	Figurski and Helinski (1979)
pMo130-Tel^R^	Suicide plasmid, xylE+, sacB+, KmR, Donor of tellurite resistance cassette (telR)	Amin et al. (2013)
pGEM-sacB	*sacB* cloned into pGEM-T, Amp^R^	Hu et al. (2021)
pGEM-xcpW	xcpW upstream -telR-xcpW downstream, joined with pGEM-sacB; TelR, Amp^R^	This study
pMo130-comA	comA upstream -telR-comA downstream, joined with ori, oriT and sacB of pMo130	This study
pMo130-xcpW	xcpW upstream -telR-xcpW downstream, joined with ori, oriT and sacB of pMo130	This study
pGEM-sacB-△pilN	pilN upstream-kan-pilN downstream cloned into pGEM-sacB; Kan^R^, Amp^R^	Hu et al. (2021)
pEASY-T1	TA cloning vector, Amp^R^, Kan^R^	TransGen
pIS26	IS26 cloned into pEASY-T1, Kan^R^	This study
*A. baumannii* strains		
HN85	Wild-type clinical MDR isolate, ST2 (Pasteur)	Lab stock
HN85_ET*^xcpW^*	Cointegration of HN85 and electroporated pGEM-xcpW	This study
HN85_ET*^comA^*	Cointegration of HN85 and electroporated pMo130-comA	This study
HN85_SR*^xcpW^*	Sucrose-resistant subcolony of HN85_ET^xcpW^	This study
HN85_SR*^comA^*	Sucrose-resistant subcolony of HN85_ET^comA^	This study
HN85 ∆*comA*	HN85 with deletion in *comA* operon	This study
HN85 ∆*xcpW*	HN85 with deletion in xcpW operon	This study
HN85_NT	grown on tellurite plate without plasmid insertion after electroporation	This study
W068	Wild-type sensitive clinical isolate, ST338 (Pasteur)	Lab stock
W068_IS*26*	Cointegration of W068 and electroporated pIS26	This study
W068_IS*26*_S	W068 with one copy of IS26 insertion	This study

### Construction of plasmids and mutant strains

A list of plasmids used in this work is shown in Table 1. All derivative plasmids were constructed using a seamless cloning strategy based on overlap extension PCR techniques (pEASY®-Basic Seamless Cloning and Assembly Kit, TRAN). The primers used throughout this study are provided in Supplementary Table S1. Approximately 800–1,000 bp of flanking regions surrounding the target genes were amplified using appropriate primers designed to delete most sequences from the target genes while preserving only the final 25–48 nucleotides at the 3′ end (Supplementary Table S1). The pMo130-Tel^R^ vector served as a PCR template for *telR* amplification, while the vector backbone for pGEM-sacB and pMo130-Tel^R^ were amplified using primers containing complementary regions aligned with those flanking the target genes.

The transfer of the plasmids into *A. baumannii* isolates was accomplished through electroporation, natural transformation, or conjugation methods as previously described (Biswas, 2015; Hu et al., 2019), followed by selection on LB plates supplemented with potassium tellurite (30 mg/L) or kanamycin (50 mg/mL). Resulting transformants containing transferred plasmids were verified through PCR using primers tel-NF/NR for *TelR* cassette detection or KF/KR for *kanR* marker identification after undergoing at least three cycles of re-culture. The position of plasmid insertion was verified through PCR and sequencing utilizing appropriate primers located outside the homologous region and within the selection markers (Supplementary Table S1). For transformants exhibiting correct plasmid integration, final knockout mutants underwent counter-selection on LB agar supplemented with 10% sucrose and tellurite (30 mg/L). The gene deletions were ultimately confirmed by PCR and sequencing.

### Whole genome sequencing and analysis

A hybrid short- and long-read based- whole genome sequencing was performed to construct the complete genome of the wild strain HN85, W068 and several transformants (Table 1). Briefly, short-read sequencing data were generated by Illumina HiSeq PE150 double-end sequencing strategy. Long-reads sequencing data were obtained from the Oxford Nanopore MinION platform in accordance with the manufacturer’s instructions. A hybrid assembly was performed utilizing Unicycler (Wick et al., 2017).

The prediction and annotation of genome sequences were performed using Prokka (Seemann, 2014). IS elements were identified through ISFinder, following current criteria for defining isoforms, which required sharing >98% amino acid similarity and/or >95% nucleotide identity with any other IS in the database (Siguier et al., 2006). Whole genome alignment among strains was constructed using Mauve v2.4.0 progressive alignment (Darling et al., 2004). A total of 931 complete genomes of *A. baumannii* were download from NCBI genome database, and the presence as well as diversity of IS*26* within these genomes was analyzed via BLAST analysis (Jia et al., 2017). Only intact copies of IS*26* were included in the distribution statistics. MLST was performed according to Pasteur’s scheme^1^ followed by eBURST analysis to assess the population structure among selected genomes. A clonal complexes (CC) is defined as a set formed by a founder ST along with its single locus variants (Diancourt et al., 2010).

### Natural transformation assay

Bacteria were tested for natural transformation as described previously (Hu et al., 2019). The donor DNA pOri was a shuttle-plasmid constructed by cloning the PCR product of the replication origin region of pWH1266 into pCR-Blunt II-TOPO. The zeocin resistance cassette of pCR-Blunt II-TOPO was used as the selectable marker.

### Data availability

The genome sequences of HN85, W068, HN85_ET*^xcpW^*, HN85_ET*^comA^*, HN85_ NT, HN85_SR*^xcpW^* and W068_IS*26*_S have been deposited in GenBank (BioProject PRJNA1264468) under accession numbers CP194389, CP194656-CP194660, CP194388, CP194387, CP194385, CP194386 and CP194651-CP194655.

## Results

### Distribution of IS elements in W068 and HN85

In our preceding gene knockout studies involving the susceptible clinical strain *A. baumannii* W068, all electroporated suicide plasmids were successfully integrated into the target site via homologous recombination (Hu et al., 2021). However, such integration was not achieved in isolate HN85 due to recruitment by IS elements (details provided below). Genome sequencing revealed that the chromosomes of HN85 and W068 are 3,975,818 bp and 3,735,903 bp in length, respectively. The W068 strain contains four plasmids measuring 13,276 bp, 10,295 bp, 9,656 bp, and 3,861 bp.

**Figure 1:**
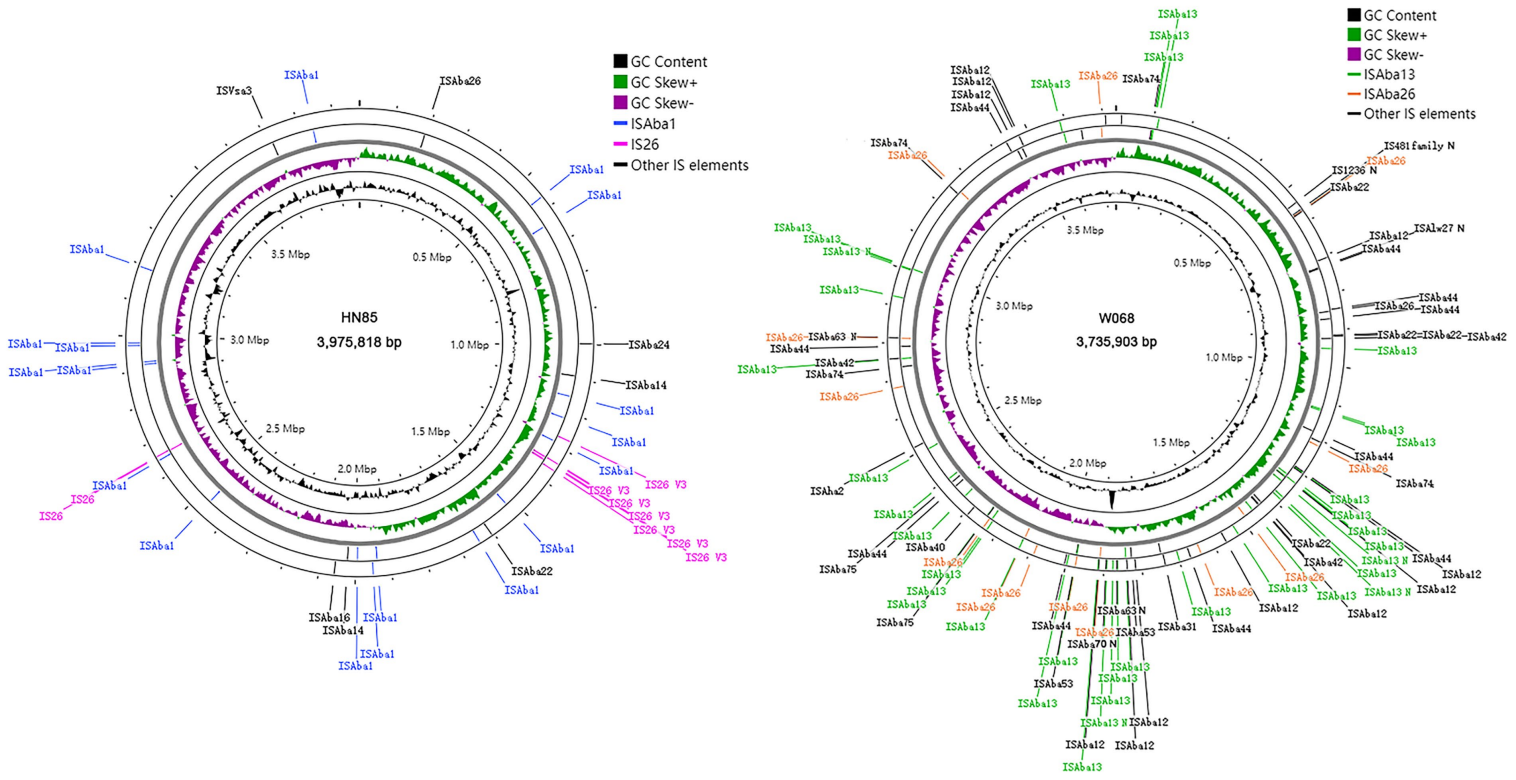
Distribution of IS elements in HN85 and W068 genomes. Circles display (from outside in order of) (i, ii) IS elements in the reverse and forward strand respectively; (iii) G-C skew; (iv) GC content. The incomplete IS elements in W068 are marked with the suffix ‘N’.

Utilizing ISFinder BLAST followed by manual examination, we identified a higher density of insertion sequence (IS) elements in W068, with 100 copies, significantly exceeding the average estimate of 33 IS copies per *A. baumannii* genome by Adams et al. (2016). In contrast, HN85 has only 35 copies (Figure 1). Some IS copies exhibited truncated or frameshift mutations (Table 2). We identified 12 intact ISs totaling 90 copies in W068 and eight intact ISs accounting for 34 copies in HN85 (Table 2). Only IS*Aba26* and IS*Aba22* were present in both genomes.

**Table 2:** Characteristics and distribution of IS elements in HN85 and W068.

IS	IS family	Group	Non-*Acinetobacter* distribution	HN85		W068	
				Complete	Incomplete	Complete	Incomplete
IS*Aba1*	IS*4*	IS*10*	Y	18			
IS*26*	IS*6*	-	Y	9	1		
IS*Aba14*	IS*3*	IS*150*	Y	2			
IS*Aba16*	IS*66*	-	NO	1			
IS*Aba24*	IS*66*	-	Y	1			
IS*Aba26*	IS*256*	-	NO	1		14	
IS*Aba22*	IS*3*	IS*3*	NO	1		4	
IS*Vsa3*	IS*CR*	-	Y	1			
IS*Aba13*	IS*5*	IS*903*	NO			33	4
IS*Aba44*	IS*630*	-	NO			12	
IS*Aba12*	IS*5*	IS*903*	NO			11	
IS*Aba74*	IS*5*	IS*903*	NO			5	
IS*Aba42*	IS*256*	-	Y			3	
IS*Aba31*	IS*5*	IS*427*	NO			2	
IS*Aba53*	IS*5*	IS*903*	NO			2	
IS*Aba75*	IS*3*	IS*150*	NO			2	
IS*Aba40*	IS*5*	IS*903*	NO			1	
IS*Aha2*	IS*5*	IS*903*	NO			1	
IS*Aba63* ^a^	IS*3*	IS*51*	NO				2
IS*Aba70* ^b^	IS*1202*	IS*Aba32*	NO				1
IS*481* family ^c^	IS*481*		NO				1
IS*1236* ^c^	IS*3*	IS*3*	NO				1
IS*Alw27* ^d^	IS*3*	IS*150*	NO				1
SUM				34	1	90	10

a one sequence is incomplete with frameshift; the other sequence has internal STOP codon and frameshift.

b a mutated sequence with a codon STOP in frame.

c a mutated sequence with internal STOP codon and unexpected frameshift.

d an incomplete sequence, missing N-terminus.

BLAST analysis indicated that most IS elements identified in strain HN85 are also present in various non-*Acinetobacter* Gram-negative bacteria (Table 2), including the three most prevalent elements: IS*Aba1*, IS*26*, and IS*Aba14*. Two novel ISs discovered in W068 have been designated as IS*Aba74* (IS*5* family) and IS*Aba75* (IS*3* family) based on submissions to the ISFinder database. These elements are also present in several other strains of *A. baumannii* and *Acinetobacter* species; however, no high-homology counterparts were identified outside this genus.

**Figure 2:**
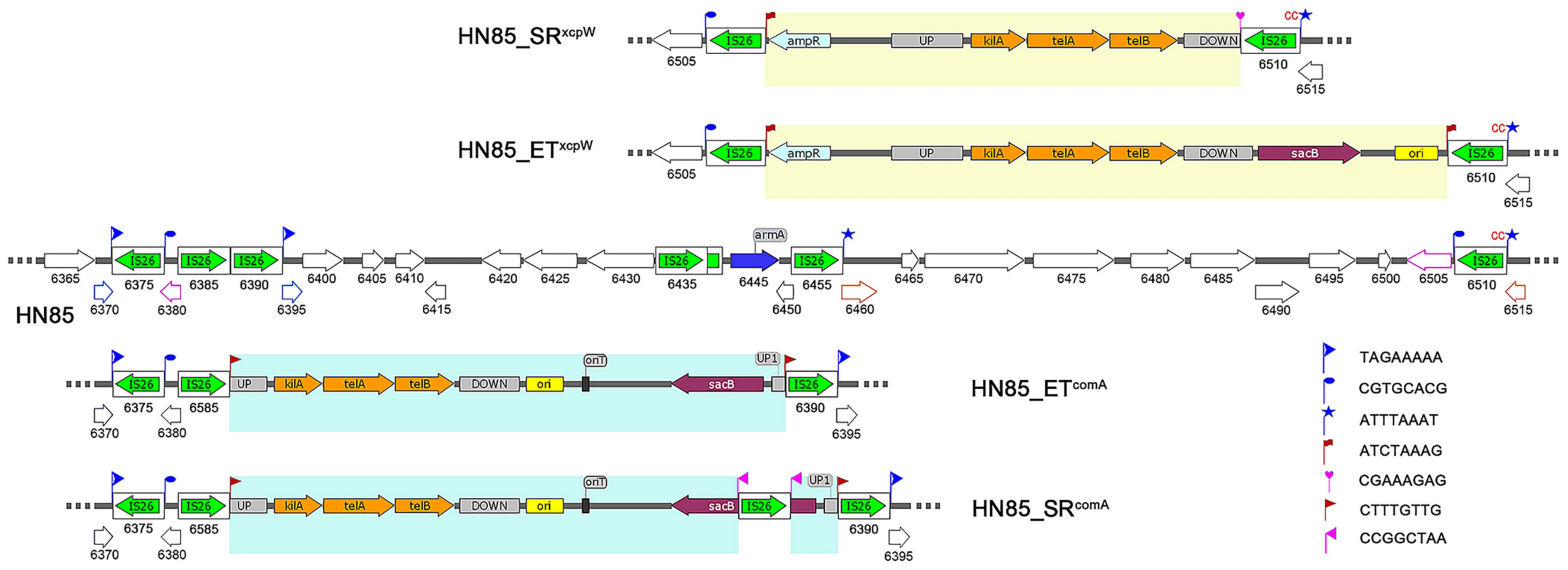
Schematic representation of IS26-mediated plasmid integration and remodeling in derivatives of *A. baumannii* HN85 genome. IS26 elements are depicted as white rounded boxes, with a green arrow showing the orientation and extent of the tnp26 transposase gene. The wild-type HN85 chromosome is illustrated in the center, with each gene’s locus_tag formatted as “ACRRTD_” followed by the corresponding number indicated below it. Pseudogenes resulting from the same ORF interruptions represented by lines of corresponding colors within the HN85 genome. Genes in the derivatives of HN85 are labeled according to their counterparts in HN85. TSDs are shown as flags. Newly generated TSDs are highlighted in red and pink. The sequences of inserted plasmids are highlighted in yellow and blue. Plasmid features include: UP, upstream region of the target gene; DOWN, downstream region of the target gene; ori, ColE1 origin for efficient plasmid replication in *E. coli*; kilA, telA and telB, tellurite resistance operon; sacB, counter-selection marker selected by sucrose.

Isolate HN85 contains one truncated copy and nine intact copies of IS*26*, with seven clustered within a specific region (see Figures 1, 2). Most non-IS*26* genes located within this IS*26*-rich region are conserved compared to those found in W068, except for the aminoglycoside resistance gene *armA* (Figure 2). Six distant pseudogenes correspond to three genes distributed throughout this region, which were indicated by different colored lines in Figure 2. Although TSDs are commonly considered a fingerprint of transposition events, no single copy of IS*26* was observed flanked by a matching pair of TSDs. However, identical TSDs were detected at one end of two distinct IS*26* copies (marked by blue flags in Figure 2), with one copy containing two additional cytosine nucleotides. For IS*26* transposition, TSDs are typically located on both sides of the incoming replicon (Harmer and Hall, 2024); however, the three fragments located between these identical TSDs exhibit mutual overlap, suggesting possibilities for multiple sequence reversals post-IS*26* insertion.

### IS*26*-mediated integration of electroporated plasmids

In our previous studies, we constructed 33 single-gene knockout mutants in W068 (Hu et al., 2021). We hypothesize that replacing the kanamycin resistance marker (*kanR*) in suicide vectors for W068 with *telR* will facilitate gene deletion in MDR isolates using the same knockout protocol. Plasmid pGEM-*xcpW* was designed as such a vector to knock out the T2SS minor pseudopilus assembly prime complex competent protein XcpW (Table 1). After electroporating this plasmid into MDR isolate HN85, numerous colonies grew on tellurite selection plates; however, only one exhibited *telR* amplification, designated HN85_ET*^xcpW^*. But PCR with primers located outside the *xcpW* flanking region yielded a product length identical to that of the wild type, indicating the integration occurred outside the target position. Genome sequencing further revealed that the insertion plasmid was located adjacent to the last IS*26* copy (ACRRTD_06510) within the aforementioned IS*26*-rich region and contained an additional copy of IS*26* on its opposite side, thereby forming a PCT structure (Figure 2), which represents the classic architecture produced by the “copy in” transposition mechanism of IS*26* (Harmer et al., 2020). The resulting TSDs were found at their expected locations and are marked with red flags in Figure 2.

We also constructed two oriT-containing knockout plasmids: pMo130-*xcpW* and pMo130-*comA* (Table 1). Similarly, most colonies that grew on tellurite selection plates after electroporation with pMo130-*comA* did not exhibit detection of the plasmid marker *telR*. The plasmid integration transformant was designated HN85_ET*^comA^*. Genome sequencing revealed that the plasmid was integrated at another location within the IS*26*-rich region, surrounded by a matching pair of TSDs and two directly oriented copies of IS*26* (Figure 2). In contrast to HN85_ET*^xcpW^*, no additional copy of IS*26* was identified in HN85_ET*^comA^*. The original sequence at the insertion site contained three closely linked copies of IS*26*, leading us to hypothesize that homologous recombination occurred during or after plasmid integration, leading to the loss of one IS*26* copy.

### Genome reorganizations mediated by IS*26*

To elucidate the mechanism underlying tellurite resistance observed in non-plasmid-integrated colonies grown on selection plates, we selected one colony (HN85_NT) for genome sequencing. A low-affinity inorganic phosphate transporter gene (*pitA*) was found disrupted by a new IS*26* insertion, causing an inversion within a 1,501,774 bp segment between the first copy of IS*26* within the IS*26*-rich region and this newly inserted copy.

To evaluate the response of these mutants to survival stressors, we cultured both HN85_ET*^xcpW^* and HN85_ET*^comA^* in LB broth supplemented with 10% sucrose and 30 mg/L potassium tellurite. The resulting sucrose-resistant subclones were designated as HN85_SR*^xcpW^* and HN85_SR*^comA^*. PCR analysis and sequencing revealed insertional inactivation and deletion of the counter-selection marker *sacB*, induced by IS*26* transposition, in HN85_SR*^comA^* and HN85_SR*^xcpW^*, respectively (Figure 2). We hypothesize that an intermediate form may exist between HN85-ET*^xcpW^* and HN85_SR*^xcpW^*, which contains a novel IS*26* insertion in the downstream homologous region of the integrated plasmid within HN85_ET*^xcpW^.* This intermediate was subsequently resolved via homologous recombination, leading to the loss of the newly inserted IS*26* copy along with a DNA segment located between two IS*26* copies, which included a pre-existing TSD (Figure 2), thus yielding HN85_SR*^xcpW^*. For HN85_SR*^comA^*, the inactivation of *sacB* by IS*26* transposition was confirmed by PCR sequencing, as the PCR product length exceeded that of the control. However, PCR is susceptible to template switching (Odelberg et al., 1995). Given that classic transposition structures for IS*26* are flanked by directly-oriented IS*26* elements, there exists a risk of missing amplification of the internal sequence between IS*26* copies due to template switching. Nevertheless, even in such cases, it remains sufficient to confirm the insertional inactivity of *sacB* by IS*26* transposition.

### Deletion of *comA* and *xcpW* by natural transformation and conjugation

Plasmids pMo130-*comA* and pMo130-*xcpW* were also introduced into HN85 via natural transformation and conjugation, respectively. After overnight cultivation, numerous colonies emerged on the tellurite selection plates. Most selected colonies exhibited amplification of *telR*; primers outside the flanking regions of *comA* and *xcpW* showed no amplifications, confirming plasmid integration since their sizes (8,833 bp and 8,998 bp) exceeded the Taq polymerase’s amplification limits used in this study. After counterselection with sucrose, we ultimately achieved in-frame deletions for both *comA* and *xcpW*.

Our previous research has confirmed that *comA* and *xcpW* are essential for the natural transformation of strain W068 (Hu et al., 2021). However, the deletion of *xcpW* did not impact the transformation capability in strain HN85. In contrast, knockout mutants lacking *comA* exhibited a complete loss of transformability.

### Distribution of IS*26* in *Acinetobacter baumannii* genomes

To investigate the distribution patterns of IS*26* within *A. baumannii* genomes, we analyzed 931 complete genomes from 43 countries deposited in Genbank^2^ (as of June 10, 2025). Detailed information about these selected genomes is provided in Supplementary Table S2. Several minor variants of IS*26* have been identified and designated as IS*26* v1-v4 based on relevant references (Harmer et al., 2021). These variants differ by one to three nucleotides, leading to single amino acid changes within the transposase. Three of these variants are listed in the ISFinder database as IS*15DI*, IS*15DII*, and IS*15DIV*. Reports indicate that the G184N substitution in Tnp26 of both IS*26* v1 and v3 exhibits enhanced activity levels (Pong et al., 2019). All instances of transposition observed in this study were attributed to IS*26* v3. HN85 contains seven copies of IS*26* v3 but only two copies of wild-type IS*26*; thus, it is challenging to exclude potential effects arising from the high copy numbers. For clarity, we collectively refer to all minor variants as IS*26*.

MLST analyses identified 154 sequence types (STs) among the 931 genomes. Intact forms of IS*26* were present in approximately 74.97% (698/931) of the samples, corresponding to 69 distinct STs. The majority (89.4%, 624/698) carried only chromosomal copies of IS*26*, while those solely carried by plasmids accounted for just 7.4% (Supplementary Table S3). In contrast, BLAST analysis against the core nucleotide database of *A. pittii* and *A. nosocomialis* revealed only nine and six plasmids carrying IS*26*, respectively; no chromosomal carrying IS*26* were detected in these closely related species.

The 154 STs were grouped into 21 CCs and 65 singletons through eBURST analysis (Supplementary Table S3). CC1, CC2, ST229, CC15, CC79, CC78, CC25, CC23 and CC85 were reported to belong to nine known international clonal lineages IC1-IC9 (Shelenkov et al., 2023). IS*26* was detected in isolates from each lineage at frequencies of 67.9, 99.8, 100, 75, 91.7, 88.9, 28.9, 39.3, and 12.5%. A total of 54.8% (510/931) of the genomes were classified as belonging to IC2. Notably, each genome within this group contained chromosomal IS*26* with copy numbers ranging from 1 to 21 (average: 5). However, two strains (NIPH17_0019 and VB1190) exhibited frameshift mutations across all chromosomal IS*26* copies (Supplementary Table S2), thus leading to their classification as lacking chromosomal IS*26* in Supplementary Table S3. The prevalence of IS*26* among IC2 strains is even higher than that observed for IS*Aba1*—the most commonly encountered IS elements in *A. baumannii* (505/510, Supplementary Table S4).

### IS*26* activities in W068

Strain HN85 contains nine copies of IS*26*, raising the question of whether the recruitment of suicide plasmids is solely due to increased opportunities from these multiple copies. The last copy in the IS*26*-rich region was amplified and cloned into pEASY-T1, resulting in plasmid pIS*26*. This plasmid shares no homologous regions with the W068 genome, except for 84 bp and 20 bp fragments flanking IS*26*. By electroporation, pIS*26* was introduced into W068 yielding kanamycin-resistant transformants designated W068_IS*26*. To promote excision of cointegrates while retaining only a single copy of IS*26* but eliminating entire plasmid fragments, W068_IS*26* was recultured without kanamycin selection pressure. Kanamycin resistance loss occurred at a high frequency (100%, *n* = 20); however, only one out of 20 tested kanamycin-sensitive colonies, designated W068_IS*26*_S, retained an intact copy of IS*26*. Sequencing revealed that this copy remained inserted within the 10,295 bp plasmid by interrupting an ORF (ACRRTB_18205) encoding a hypothetical protein.

**Figure 3:**
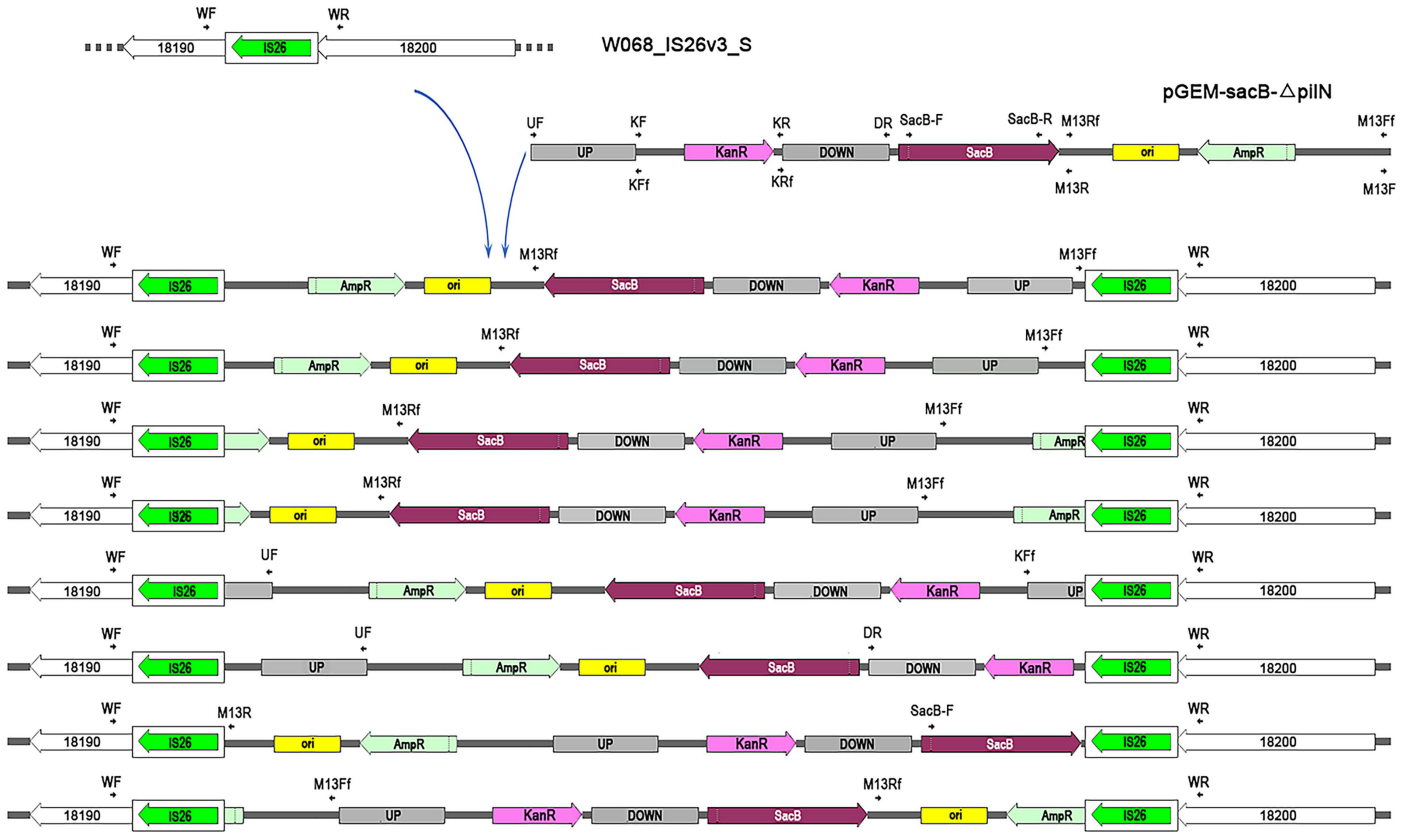
Schematic representation of IS26-mediated plasmid integration in *A. baumannii* W068IS26S genome. The insertion position of IS26 for W068IS26S is indicated above the left arrow, with each gene’s locustag formatted as “ACRRTF” followed by the corresponding number. The unique integration of pGEM-sacB-∆pilN across eight electroporation transformants are illustrated in the figures beneath the arrow. IS26 elements are shown as white rounded boxes, with a green arrow indicating the orientation and extent of the tnp26 transposase gene. Primers utilized for confirming plasmid insertion are denoted by parallel arrows. Plasmid features include: UP, upstream region of pilN; DOWN, downstream region of pilN; ori, ColE1 ori for efficient plasmid replication in *E. coli*; KanR, kanamycin resistance cassette; AmpR, ampicillin resistance cassette; sacB, counter-selection marker selected by sucrose.

The plasmid pGEM-sacB-∆*pilN* was selected for electroporation into W068_IS*26*_S, which had previously been utilized for a successful knockout of *pilN* in the wild-type W068 (Hu et al., 2021). PCR confirmed the presence of the selection marker *kanR* in all tested colonies grown on selection plates. We selected 16 colonies for further examination of insertion sites. PCR using primers outside the flanking region of *pilN* showed that 8 colonies had amplification lengths identical to wild-type W068, suggesting plasmids were inserted outside the target region. To confirm this, we performed PCR with IS*26* flank primers (WF and WR) paired with plasmid-specific primers followed by sequencing, which indicated that all eight colonies integrated the plasmid within the PCT structure of IS*26* (Figure 3). The integrated plasmids were interrupted at different positions and inserted in various orientations, indicating a random pattern in target site selection by IS*26*.

To evaluate how W068_IS*26*_S responds to stress, electroporation without additional DNA was conducted. Following a one-hour incubation at 37 °C with shaking to promote recovery and growth, both treated cells and an equal amount of untreated W068_IS*26*_S cells were plated onto tellurite-containing agar. After overnight cultivation, only the samples subjected to electroporation exhibited colony growth. Genetic analysis through PCR and sequencing confirmed that approximately one-third of tellurite-resistant colonies (7 out of 23) exhibited insertional interruptions in *pitA* due to IS*26* transposons.

## Discussion

Allelic exchange mutagenesis is an efficient method for bacterial genome editing. This approach is particularly effective in susceptible clinical strains of *A. baumannii*. However, when applied to the MDR IC2 clinical strain HN85, electroporation of suicide plasmids consistently resulted in their integration into adjacent regions of IS*26* rather than insertion at target sites through homologous recombination. This precludes the possibility of deleting target genes through a subsequent recombination event. The recruitment of plasmids was facilitated by the specialized co-integrate movement mechanism inherent to IS*26*. The replicative transposition of IS*26* was mobilized, allowing for targeting and ultimate integration of the suicide plasmid into the chromosome. This integration process can occur independently of homologous recombination (Harmer and Hall, 2016), thereby enabling capture of foreign DNA without requiring homology. Furthermore, when counter-selection was applied to the plasmid cointegrates, IS*26* exhibited remarkable “clean-up” capabilities by deleting or interrupting harmful *sacB* while preserving beneficial *telR* cassettes. Thus, IS*26* functions not only as a DNA capturer but also a “fixer.”

The high activity observed for IS*26* in HN85 may be attributed to its high copy number. After introducing a single copy of IS*26* into sensitive strain W068, plasmid integration was also influenced. The electroporation-mediated transfer of suicide plasmids no longer resulted solely in integration at target regions. The recruitment catalyzed by Tnp26 occurred with frequencies comparable to those associated with homologous recombination, consistent with findings reported by Harmer and Hall (2016). Consequently, we speculate that IS*26* at least doubles the probability of successful transformation for its host strain when exposed to exogenous double-stranded DNA (dsDNA), such as DNA transported by outer membrane vesicles. In strains harboring multiple IS*26* PCT structures, such as HN85, TU excision followed by subsequent replicative transposition may occur more frequently than in W068_IS*26*_S. This enhanced frequency would significantly improve their capacity to acquire exogenous genes, leading to consistent recruitment of electroporated plasmids by IS*26* within HN85.

In the case of HN85, following electroporation, a higher number of cells opted to mobilize their own genetic elements, such as the insertional inactivation of *pitA*, rather than integrate suicide plasmids when subjected to selection pressure from tellurite. Reports indicate that tellurite mainly enters *Escherichia coli* via the PitA phosphate transporter (Elias et al., 2012). Tellurite exerts toxic effects only once it is inside the cell; therefore, inactivation of *pitA* can significantly reduce intracellular tellurite concentration, leading to high tellurite tolerance. This mechanism should be more efficient than integrating a suicide plasmid and expressing its selection markers to mitigate the toxicity of tellurites, which requires more metabolic investment. This may explain why false-positive colonies frequently emerged during screening for plasmid integration variants of HN85 using tellurite plates. Although insertional inactivation of *pitA* was also observed in W068_IS*26*_S, it occurred solely post-electroporation. There is currently limited understanding regarding the regulation of IS*26* transposition. We hypothesize that electroporation may induce an SOS response that subsequently stimulates IS*26* transposition through an unknown mechanism.

HN85 and W068 exhibit distinct IS profiles. In HN85, the two predominant elements, IS*Aba1* and IS*26*, account for 79.4% of total IS copies and both contribute to its MDR phenotype. As a DNA capturer, IS*26* has incorporated the aminoglycoside resistance gene *armA* (Doi et al., 2016). The insertion of IS*Aba1* upstream of *ampC* along with two *bla*_OXA-23_ copies, provides a robust promoter that confers clinical resistance to cephalosporins and carbapenems, respectively (Segal et al., 2007; Hamidian and Hall, 2013). In contrast, the genome of W068 predominantly contains *Acinetobacter* genus-specific IS elements from the IS*5* family, which are absent in HN85. The two most prevalent IS elements in W068—IS*Aba13* and IS*Aba26* (with 33 and 14 copies respectively) —were reported to have no effect on adjacent gene expression (Wright et al., 2017). The extensive accumulation of IS insertions observed in W068 is not an attribute unique to susceptible *A. baumannii* strains; for example, ATCC17978 and ATCC19606 possess fewer than 10 intact IS insertions. Although these insertions do not confer a resistance advantage in W068, they may facilitate genome remodeling that aids adaptation to environmental pressures. Fortunately, none of the IS elements in W068 interfered with plasmid transformation in our previous study.

Although the genetic backgrounds are distinct, IS*26* was consistently mobilized preferentially in HN85 and W068_IS*26*_S compared to other IS elements. This strong transposition activity may explain the widespread distribution of IS*26* among Gram-negative bacteria. In summary, the role of IS*26* in *A. baumannii* strains can be likened to that of a security guard: it responds promptly to attacks or stressors, effectively neutralizing threats (by inserting into or deleting deleterious genes), and swiftly acquiring resources (such as foreign dsDNA) to ensure survival. The most prevalent and resistant clonal lineage within *A. baumannii* corresponds with the most extensively disseminated clones harboring IS*26*, highlighting its significant contribution to the evolution and success of IC2. It is plausible that acquisition events involving IS*26* occurred early in the evolutionary history of IC2. Subsequently, the strong recruitment and transposition capabilities of IS*26* played a significant role in the development of multidrug resistance and endowed *A. baumannii* with remarkable genome remodeling abilities necessary for adapting to environmental stresses.

Compared to conjugation and natural transformation, electroporation is a more commonly employed method for transferring exogenous DNA due to its lack of restrictions on plasmids or recipient cells. However, particular attention must be given to the active recruitment by IS*26* after electroporation, not only in *A. baumannii* but also across Gram-negative bacteria where IS*26* is widely disseminated. When DNA is transferred through conjugation or natural transformation, only a single strand of DNA can be incorporated into recipient cells (Chen and Dubnau, 2004; de la Cruz et al., 2010), which minimizes targeting by IS elements and facilitates integration at target sites via homologous recombination.

The deletion of *xcpW* showed varying impacts on natural transformation between HN85 and W068 strains. The W068 strain exhibits no twitching motility and lacks visible extracellular type IV pili filaments, which are required for DNA uptake. XcpW may play a crucial role in facilitating DNA uptake in this strain by promoting the formation of a specialized competence pseudopilus (Hu et al., 2021). In contrast, HN85 exhibits twitching motility, indicting its type IV pili are functional; consequently, it does not necessitate an alternative specialized competence pseudopilus for effective DNA uptake. Therefore, deletion of *xcpW* had no discernible impact on competence levels in HN85. This suggests that the mechanism involved in DNA uptake during natural transformation may be more complex than previously estimated; subtle differences exist between various *A. baumannii* strains.

## Data Availability

The datasets presented in this study can be found in online repositories. The names of the repository/repositories and accession number(s) can be found at: https://www.ncbi.nlm.nih.gov/genbank/, PRJNA1264468.

## Footnotes

^1^ http://www.pasteur.fr/mlst

^2^ https://www.ncbi.nlm.nih.gov/genbank/
