# Supplementary material for: Impact of IS26 mobilization on genetic manipulation of multidrug-resistant Acinetobacter baumannii
Source: Front Microbiol. 2025 Oct 8;16:1689239. doi: 10.3389/fmicb.2025.1689239 (PMC12540391; doi:10.3389/fmicb.2025.1689239)
Supplement: Supplementary file 1 [file Data_Sheet_1.pdf]

Table S1. primers used in this study

Primer	Sequence	Location
**comA UF**	TACCGAGCTCGGATCCACTTGGCAACTATTTATCCGG	upstream region of *comA*
**comA UR**	GGTCGACGGATCCCCGGAATGGCAAAATTAAAGCCCAACT	
**comA DF**	CGAAGCAGCTCCAGCCTACACTTTACATGACCGACGTTTGG	downstream region of *comA*
**comA DR**	GGCAAGTTAGTTACGCAGCTTTACATGACCGACGTTTGG	
**comA-WF**	ATCTGTTCGATGCCTATTTTGT	the outer region of the homologous regions of *comA*
**comA-WR**	TCATTTTGTGGTTTTGGTGG	
**comA-F**	TCTATTGGGGTGGATTGGC	in frame region of *comA*
**comA-R**	GGTTGATAATAATGTTCGTAGTGC	
**xcpW UF**	TACCGAGCTCGGATCCAGGCATTAGCCTTACCCAAAC	upstream region of *xcpW*
**xcpW UR**	GGTCGACGGATCCCCGGAATTCATTATTTTGCTTTCACTGGAT	
**xcpW DF**	CGAAGCAGCTCCAGCCTACACTCAGGGTGACTTATCGCTTTC	downstream region of *xcpW*
**xcpW DR**	GGCAAGTTAGTTACGCAGCGCACTAGTAAACTGTCGCTTCC	
**xcpW-WF**	CATGGAGCTGCTGCAATTCT	the outer region of the homologous regions of *xcpW*
**xcpW-WR**	ATGGAGCCATGCTTCTTGAA	
**xcpW-F**	CTCGATTAACTCGCGCCTC	in frame region of *xcpW*
**xcpW-R**	ATTTTGTGACTTCTTGCGGAG	
**pilM-F**	GAGCTCTCTGTCAAGAACGGT	the outer region of the homologous regions of *pilN*
**pilP-R**	AATCTGCTGATCCGGTGTCT	
**Tel-Fd**	ATTCCGGGGATCCGTCGACCCAGCAATGGATATCAGCCGT	whole *telR* cassette of pMo130-TelR
**Tel-Rd**	GTGTAGGCTGGAGCTGCTTCGCGAGCAGAAAGTCAAAAGCC	
**tel-NF**	CCTTCTTGGCGTCCACATC	in frame region of *telA*
**tel-NR**	TCATACGGACTCCTGTTGGG	
**KF**	ATTCCGGGGATCCGTCGACC	kanR of pGEM-sacB-△pilN
**KR**	GTGTAGGCTGGAGCTGCTTCG	
**KFf**	GGTCGACGGATCCCCGGAAT	reverse complement of KF
**KRf**	CGAAGCAGCTCCAGCCTACAC	reverse complement of KR
**M13F**	TGTAAAACGACGGCCAGT	The vector backbone of pGEM-sacB-△pilN
**M13R**	CAGGAAACAGCTATGACC	The vector backbone of pGEM-sacB-△pilN
**M13Ff**	ACTGGCCGTCGTTTTACA	The vector backbone of pGEM-sacB-△pilN
**M13Rf**	CATGGTCATAGCTGTTTCCTG	The vector backbone of pGEM-sacB-△pilN
**UF**	ATGCTTTGATGCCTTTACCC	upstream region of pilN in pGEM-sacB-△pilN
**DR**	AATCGGTGCTGGTTCAATAG	downstream region of pilN in pGEM-sacB-△pilN
**AKD-R**	GCTGCGTAACTAACTTGCCATC	the vector backbone of pGEM-sacB
**19AKDs-F**	TGGATCCGAGCTCGGTAGCCCAATTCGCCCTATAGTG	
**pUC19sacB-F**	GCTGCGTAACTAACTTGCCACAGAATCAGGGGATAACGC	the vector backbone of pMo130-TelR (the origin of replication, *oriT* and *sacB*)
**pUC19sacB-R**	TGGATCCGAGCTCGGTACGCCTTCTTGACGAGTTCTT	
**IS26-Fc**	TCAGTAATTGGACGTGCACG	the last IS*26* of IS*26*-rich region, including 84 bp and 20 bp of the flank sequence
**IS26-Rc**	CCATTCTCATGTGTTAGATATCTTTG	
**IS26-F**	AACTTATCATCCCCTTTTGCTG	in frame region of IS*26*
**IS26-R**	GCAAAGTTAGCGATGAGGCA	
**sacB-F**	TGCGTAACTAACTTGCCATCT	*sacB*
**sacB-R**	GGCATTTTCTTTTGCGTTTT	
**pitA-R**	CAAACAGATGGTTGCAGGAA	in frame region of *pitA* (12-1593 of the 1632 bp ORF)
**pitA-F**	TCTACCCCCTGTTTCGGATT	
**WF**	CCTCATTATCTTGAATCCATCC	flank region of IS*26* in W068_IS26_S
**WR**	TCAAGAGAAACGTCAATACCCT	
