# Supplementary material for: Impact of IS26 mobilization on genetic manipulation of multidrug-resistant Acinetobacter baumannii
Source: Front Microbiol. 2025 Oct 8;16:1689239. doi: 10.3389/fmicb.2025.1689239 (PMC12540391; doi:10.3389/fmicb.2025.1689239)
Supplement: Supplementary file 2 [file Data_Sheet_2.pdf]

Table S2. Distribution of IS*26* in *A. baumannii* genomes

Country	CC	ST(Pasteur)	Accession	strain	Frameshifted	Truncated	Complete
Poland	CC2	ST2(2-2-2-2-2-2-2)	CP121345.1	96			4
China	CC2	ST2(2-2-2-2-2-2-2)	CP096768.1	5388	1	2	8
China	CC2	ST2(2-2-2-2-2-2-2)	CP096766.1	5626		4	17
China	CC2	ST2(2-2-2-2-2-2-2)	CP096764.1	5634		2	10
China	CC2	ST2(2-2-2-2-2-2-2)	CP096762.1	5651			4
China	CC2	ST2(2-2-2-2-2-2-2)	CP096759.1	5653	1	2	9
China	CC2	ST2(2-2-2-2-2-2-2)	CP096757.1	5656	1	1	7
China	CC2	ST2(2-2-2-2-2-2-2)	CP096755.1	5663		1	7
China	CC2	ST2(2-2-2-2-2-2-2)	CP096753.1	5664		1	7
China	CC2	ST2(2-2-2-2-2-2-2)	CP096749.1	5666		1	5
China	CC2	ST2(2-2-2-2-2-2-2)	CP096747.1	5669	1	2	7
China	CC2	ST2(2-2-2-2-2-2-2)	CP096745.1	5670		2	7
China	CC2	ST2(2-2-2-2-2-2-2)	CP096742.1	5671	1		3
China	CC2	ST2(2-2-2-2-2-2-2)	CP096740.1	5672		2	11
China	CC2	ST2(2-2-2-2-2-2-2)	CP096738.1	5679			2
China	CC2	ST2(2-2-2-2-2-2-2)	CP096735.1	5683		1	7
China	CC2	ST2(2-2-2-2-2-2-2)	CP096734.1	5685			5
China	CC2	ST2(2-2-2-2-2-2-2)	CP096731.1	5689		1	7
China	CC2	ST2(2-2-2-2-2-2-2)	CP096729.1	5729		1	6
China	CC2	ST2(2-2-2-2-2-2-2)	CP096727.1	5732	2	1	8
China	CC2	ST2(2-2-2-2-2-2-2)	CP096724.1	5734		1	5
China	CC2	ST2(2-2-2-2-2-2-2)	CP096722.1	5736		1	5
China	CC2	ST2(2-2-2-2-2-2-2)	CP096720.1	5740		2	8
China	CC2	ST2(2-2-2-2-2-2-2)	CP096717.1	5741		1	6
China	CC2	ST2(2-2-2-2-2-2-2)	CP096714.1	5745		1	6
China	CC2	ST2(2-2-2-2-2-2-2)	CP096710.1	5759		1	7
China	CC2	ST2(2-2-2-2-2-2-2)	CP096707.1	5760		2	8
China	CC2	ST2(2-2-2-2-2-2-2)	CP096705.1	5761		1	6
China	CC2	ST2(2-2-2-2-2-2-2)	CP096704.1	5765		1	6
China	CC2	ST2(2-2-2-2-2-2-2)	CP096702.1	5767	1	2	8
China	CC2	ST2(2-2-2-2-2-2-2)	CP096700.1	5768	3	1	9
China	CC2	ST2(2-2-2-2-2-2-2)	CP096698.1	5769		1	9
China	CC2	ST2(2-2-2-2-2-2-2)	CP096696.1	5771	2	3	9
China	CC2	ST2(2-2-2-2-2-2-2)	CP096693.1	5773	1	1	10
China	CC2	ST2(2-2-2-2-2-2-2)	CP096692.1	5779	1		10
China	CC2	ST2(2-2-2-2-2-2-2)	CP096690.1	5839		1	7
China	CC2	ST2(2-2-2-2-2-2-2)	CP096688.1	5840	3	3	4
Mexico	CC2	ST2(2-2-2-2-2-2-2)	CP023034.1	5845		1	7
China	CC2	ST2(2-2-2-2-2-2-2)	CP096686.1	5846		1	7
China	CC2	ST2(2-2-2-2-2-2-2)	CP096684.1	5847	1		6
China	CC2	ST2(2-2-2-2-2-2-2)	CP096682.1	5955	4	1	7
China	CC2	ST2(2-2-2-2-2-2-2)	CP096681.1	6080	1	1	4
India	CC2	ST2(2-2-2-2-2-2-2)	CP045528.1	6507		1	4
Mexico	CC2	ST1544(2-2-2-2-2-129-2)	CP023031.1	7847		2	8
Mexico	CC2	ST2(2-2-2-2-2-2-2)	CP023026.1	10042		2	8
China	CC2	ST2(2-2-2-2-2-2-2)	CP104751.1	37662		2	8
USA	CC2	ST2(2-2-2-2-2-2-2)	CP107590.1	1326569			5

Country	CC	ST(Pasteur)	Accession	strain	Frameshifted	Truncated	Complete
Australia	CC2	ST2(2-2-2-2-2-2-2)	CP079743.1	4117201		1	5
Australia	CC2	ST2(2-2-2-2-2-2-2)	CP079943.1	04117201 isolate DstkR-1	1	1	4
Australia	CC2	ST2(2-2-2-2-2-2-2)	CP079944.1	04117201 isolate DstkR-2		1	5
Canada	CC2	ST2(2-2-2-2-2-2-2)	CP034242.1	09A16CRGN0014		1	6
Canada	CC2	ST2(2-2-2-2-2-2-2)	CP034243.1	09A16CRGN003B		1	6
Canada	CC2	ST2(2-2-2-2-2-2-2)	CP035186.1	11A1213CRGN008		2	7
Canada	CC2	ST2(2-2-2-2-2-2-2)	CP035185.1	11A1213CRGN055		2	7
Canada	CC2	ST2(2-2-2-2-2-2-2)	CP043419.1	11A1213CRGN064		2	7
Canada	CC2	ST2(2-2-2-2-2-2-2)	CP035184.1	11A1314CRGN088		2	7
Canada	CC2	ST2(2-2-2-2-2-2-2)	CP043418.1	11A1314CRGN089		2	8
Canada	CC2	ST2(2-2-2-2-2-2-2)	CP035183.1	11A14CRGN003		2	7
USA	CC2	ST2(2-2-2-2-2-2-2)	CP107587.1	1326527-2			5
USA	CC2	ST2(2-2-2-2-2-2-2)	CP107605.1	1326924-1			6
South Korea	CC2	ST2(2-2-2-2-2-2-2)	CP020590.1	15A34		1	7
South Korea	CC2	ST2(2-2-2-2-2-2-2)	CP020574.1	15A5			8
South Korea	CC2	ST2(2-2-2-2-2-2-2)	CP001921.1	1656-2	1		8
USA	CC2	ST2(2-2-2-2-2-2-2)	CP043469.1	18WIARLN0021			7
USA	CC2	ST2(2-2-2-2-2-2-2)	CP043462.1	18WIARLN0023			7
USA	CC2	ST2(2-2-2-2-2-2-2)	CP043455.1	18WIARLN0025			6
USA	CC2	ST2(2-2-2-2-2-2-2)	CP043452.1	18WIARLN0035			6
China, Taiwan	CC2	ST2(2-2-2-2-2-2-2)	CP131889.1	2006S08-082		1	6
China, Taiwan	CC2	ST2(2-2-2-2-2-2-2)	CP131893.1	2008C02-166	2	1	5
China, Taiwan	CC2	ST2(2-2-2-2-2-2-2)	CP131898.1	2008N08-156	1	2	6
China, Taiwan	CC2	ST195(2-2-2-2-2-1-2)	CP033516.1	2008S11-069	3	2	4
China, Taiwan	CC2	ST195(2-2-2-2-2-1-2)	CP131885.1	2008S11-069	1	2	6
China, Taiwan	CC2	ST2(2-2-2-2-2-2-2)	CP131901.1	2010C01-165		1	6
China, Taiwan	CC2	ST2(2-2-2-2-2-2-2)	CP131896.1	2010N16-194		1	5
China, Taiwan	CC2	ST2(2-2-2-2-2-2-2)	CP131903.1	2010N17-053		1	5
China	CC2	ST2(2-2-2-2-2-2-2)	CP059354.1	2014BJAB1		2	10
China	CC2	ST2(2-2-2-2-2-2-2)	CP059352.1	2014LNAB1	3	2	4
China	CC2	ST2(2-2-2-2-2-2-2)	CP059349.1	2014TJAB1			2
China	CC2	ST2(2-2-2-2-2-2-2)	CP059355.1	2016BJAB1			4
China	CC2	ST2(2-2-2-2-2-2-2)	CP059353.1	2016LNAB1	2	1	2
China	CC2	ST2(2-2-2-2-2-2-2)	CP059351.1	2018BJAB1			6
China	CC2	ST2(2-2-2-2-2-2-2)	CP059350.1	2018BJAB2			6
China	CC2	ST2(2-2-2-2-2-2-2)	CP059356.1	2018HBAB1		2	8
China	CC2	ST2(2-2-2-2-2-2-2)	CP059358.1	2018HLJAB1			4
China	CC2	ST2(2-2-2-2-2-2-2)	CP059357.1	2018HLJAB2			4
China	CC2	ST2(2-2-2-2-2-2-2)	CP059359.1	2018TJAB1			5
USA	CC2	ST2(2-2-2-2-2-2-2)	CP132214.1	2022CK-00066			5
USA	CC2	ST2(2-2-2-2-2-2-2)	CP115626.1	2022CK-00185			5
USA	CC2	ST2(2-2-2-2-2-2-2)	CP115629.1	2022CK-00337			4
USA	CC2	ST2(2-2-2-2-2-2-2)	CP124678.1	2022CK-00772			5
USA	CC2	ST2(2-2-2-2-2-2-2)	CP117728.1	2022CK-00783			5
USA	CC2	ST2(2-2-2-2-2-2-2)	CP117764.1	2022CK-00784	1		3
USA	CC2	ST2(2-2-2-2-2-2-2)	CP117762.1	2022CK-00839			5
USA	CC2	ST2(2-2-2-2-2-2-2)	CP117759.1	2022CK-00843	1		1
USA	CC2	ST2(2-2-2-2-2-2-2)	CP137927.1	2023CK-00001			3

Country	CC	ST(Pasteur)	Accession	strain	Frameshifted	Truncated	Complete
USA	CC2	ST2(2-2-2-2-2-2-2)	CP137930.1	2023CK-00003			2
USA	CC2	ST2(2-2-2-2-2-2-2)	CP131950.1	2023CK-00127			4
USA	CC2	ST2(2-2-2-2-2-2-2)	CP130921.1	2023CK-00423			5
USA	CC2	ST2(2-2-2-2-2-2-2)	CP131944.1	2023CK-00890		1	3
USA	CC2	ST2(2-2-2-2-2-2-2)	CP140434.1	2023CK-01274			3
USA	CC2	ST2(2-2-2-2-2-2-2)	CP140438.1	2023CK-01292			3
USA	CC2	N8(2-2-2-2-2-2#-2)	CP143346.1	2023CK-01486		2	7
USA	CC2	N8(2-2-2-2-2-2#-2)	CP143344.1	2023CK-01487		2	7
USA	CC2	ST2(2-2-2-2-2-2-2)	CP143348.1	2023CK-01512			3
USA	CC2	ST2(2-2-2-2-2-2-2)	CP155476.1	2024CK-00247			3
USA	CC2	ST2(2-2-2-2-2-2-2)	CP149831.1	2024CK-00250			2
USA	CC2	ST2(2-2-2-2-2-2-2)	CP155724.1	2024CK-00319			3
USA	CC2	ST2(2-2-2-2-2-2-2)	CP155458.1	2024CK-00329			4
USA	CC2	ST2(2-2-2-2-2-2-2)	CP157252.1	2024CK-00375	1		4
USA	CC2	ST2(2-2-2-2-2-2-2)	CP157249.1	2024CK-00376	1		4
USA	CC2	ST2(2-2-2-2-2-2-2)	CP157255.1	2024CK-00452			5
USA	CC2	ST2(2-2-2-2-2-2-2)	CP157480.1	2024CK-00461			6
USA	CC2	ST2(2-2-2-2-2-2-2)	CP157257.1	2024CK-00462			4
USA	CC2	ST2(2-2-2-2-2-2-2)	CP157260.1	2024CK-00536		1	4
USA	CC2	ST2(2-2-2-2-2-2-2)	CP157344.1	2024CK-00537		1	4
USA	CC2	ST2(2-2-2-2-2-2-2)	CP157342.1	2024CK-00538		1	4
USA	CC2	ST2(2-2-2-2-2-2-2)	CP166162.1	2024CK-00647			3
USA	CC2	ST2(2-2-2-2-2-2-2)	CP168455.1	2024CK-01048			4
USA	CC2	ST2(2-2-2-2-2-2-2)	CP168448.1	2024CK-01097			2
USA	CC2	ST2(2-2-2-2-2-2-2)	CP169602.1	2024CK-01224			6
USA	CC2	ST2(2-2-2-2-2-2-2)	CP169605.1	2024CK-01225			6
USA	CC2	ST2(2-2-2-2-2-2-2)	CP169608.1	2024CK-01226			6
USA	CC2	ST2(2-2-2-2-2-2-2)	CP169597.1	2024CK-01357			3
USA	CC2	ST2(2-2-2-2-2-2-2)	CP174045.1	2024CK-01384			2
USA	CC2	ST2(2-2-2-2-2-2-2)	CP174047.1	2024CK-01385			5
USA	CC2	ST2(2-2-2-2-2-2-2)	CP174050.1	2024CK-01386			2
USA	CC2	ST2(2-2-2-2-2-2-2)	CP174440.1	2024CK-01391			2
USA	CC2	ST2(2-2-2-2-2-2-2)	CP174435.1	2024CK-01459	1		4
USA	CC2	ST2(2-2-2-2-2-2-2)	CP174430.1	2024CK-01503	1		4
USA	CC2	ST2(2-2-2-2-2-2-2)	CP174432.1	2024CK-01504			5
USA	CC2	ST2(2-2-2-2-2-2-2)	CP180119.1	2024CK-01531	1		4
USA	CC2	ST2(2-2-2-2-2-2-2)	CP180115.1	2024CK-01544			5
USA	CC2	ST2(2-2-2-2-2-2-2)	CP180113.1	2024CK-01551			5
USA	CC2	ST2(2-2-2-2-2-2-2)	CP180103.1	2024CK-01557	1		4
USA	CC2	ST2(2-2-2-2-2-2-2)	CP180105.1	2024CK-01558			5
USA	CC2	ST2(2-2-2-2-2-2-2)	CP180108.1	2024CK-01559			5
USA	CC2	ST2(2-2-2-2-2-2-2)	CP180111.1	2024CK-01560			5
USA	CC2	ST2(2-2-2-2-2-2-2)	CP180101.1	2024CK-01569			2
USA	CC2	ST2(2-2-2-2-2-2-2)	CP180094.1	2024CK-01570	1		2
USA	CC2	ST2(2-2-2-2-2-2-2)	CP180096.1	2024CK-01571			6
USA	CC2	ST2(2-2-2-2-2-2-2)	CP180098.1	2024CK-01572			7
USA	CC2	ST2(2-2-2-2-2-2-2)	CP180092.1	2024CK-01662		2	5
USA	CC2	ST2(2-2-2-2-2-2-2)	CP180044.1	2024CK-01664			5

Country	CC	ST(Pasteur)	Accession	strain	Frameshifted	Truncated	Complete
USA	CC2	ST2(2-2-2-2-2-2-2)	CP180042.1	2024CK-01670			6
USA	CC2	ST2(2-2-2-2-2-2-2)	CP180038.1	2024CK-01683			5
USA	CC2	ST2(2-2-2-2-2-2-2)	CP180034.1	2024CK-01696			2
USA	CC2	ST2(2-2-2-2-2-2-2)	CP180036.1	2024CK-01697		2	5
USA	CC2	ST2(2-2-2-2-2-2-2)	CP180031.1	2024CK-01721			5
USA	CC2	ST2(2-2-2-2-2-2-2)	CP180029.1	2024CK-01738			3
USA	CC2	ST2(2-2-2-2-2-2-2)	CP181411.1	2024CK-01880			3
USA	CC2	ST2(2-2-2-2-2-2-2)	CP194118.1	2025CK-00349			6
China	CC2	ST2(2-2-2-2-2-2-2)	CP116803.1	37662RM1	1	2	7
China	CC2	ST2(2-2-2-2-2-2-2)	CP116442.1	37662RM1d21	2	2	6
China	CC2	ST2(2-2-2-2-2-2-2)	CP116801.1	37662RM2		2	8
China	CC2	ST2(2-2-2-2-2-2-2)	CP116440.1	37662RM2d21	5	2	3
South Korea	CC2	ST2(2-2-2-2-2-2-2)	CP145430.1	A20AB02			3
South Korea	CC2	ST187(2-2-2-2-2-2-43)	CP142100.1	A20AB13			1
Netherlands	CC2	ST2(2-2-2-2-2-2-2)	CP032055.1	A320 (RUH134)		1	7
unknown	CC2	ST2(2-2-2-2-2-2-2)	CP099857.1	A54R		2	9
unknown	CC2	ST2(2-2-2-2-2-2-2)	CP099858.1	A54S		1	8
unknown	CC2	ST2(2-2-2-2-2-2-2)	CP102580.1	A9844			3
China	CC2	ST2(2-2-2-2-2-2-2)	CP090182.1	AB1343		1	8
Belgium	CC2	ST2(2-2-2-2-2-2-2)	CP091368.1	AB167-VUB			5
Belgium	CC2	ST2(2-2-2-2-2-2-2)	CP091375.1	AB16-VUB		2	8
Belgium	CC2	ST2(2-2-2-2-2-2-2)	CP091366.1	AB171-VUB		1	3
Belgium	CC2	ST2(2-2-2-2-2-2-2)	CP091365.1	AB172-VUB		1	3
Belgium	CC2	ST2(2-2-2-2-2-2-2)	CP091364.1	AB173-VUB			4
Belgium	CC2	ST2(2-2-2-2-2-2-2)	CP091363.1	AB175-VUB		1	3
Belgium	CC2	ST2(2-2-2-2-2-2-2)	CP091359.1	AB180-VUB	1		4
Belgium	CC2	ST2(2-2-2-2-2-2-2)	CP091358.1	AB181-VUB		1	9
Belgium	CC2	ST2(2-2-2-2-2-2-2)	CP091357.1	AB183-VUB			6
Belgium	CC2	ST2(2-2-2-2-2-2-2)	CP091353.1	AB189-VUB			6
China	CC2	ST2(2-2-2-2-2-2-2)	CP166770.1	AB191			5
Belgium	CC2	ST2(2-2-2-2-2-2-2)	CP091352.1	AB193-VUB			6
Belgium	CC2	ST2(2-2-2-2-2-2-2)	CP091351.1	AB194-VUB			6
Belgium	CC2	ST2(2-2-2-2-2-2-2)	CP091350.1	AB212-VUB		1	6
Belgium	CC2	ST2(2-2-2-2-2-2-2)	CP091349.1	AB213-VUB			7
Belgium	CC2	ST2(2-2-2-2-2-2-2)	CP091348.1	AB214-VUB			5
Belgium	CC2	ST2(2-2-2-2-2-2-2)	CP091347.1	AB216-VUB		1	5
Belgium	CC2	ST2(2-2-2-2-2-2-2)	CP091346.1	AB217-VUB			5
Belgium	CC2	ST2(2-2-2-2-2-2-2)	CP091345.1	AB219-VUB		1	5
Belgium	CC2	ST2(2-2-2-2-2-2-2)	CP091344.1	AB220-VUB		1	5
Belgium	CC2	ST2(2-2-2-2-2-2-2)	CP091343.1	AB222-VUB			3
Belgium	CC2	ST2(2-2-2-2-2-2-2)	CP091342.1	AB224-VUB			5
Belgium	CC2	ST604(2-2-2-55-2-2-2)	CP091341.1	AB226-VUB			6
China	CC2	ST2(2-2-2-2-2-2-2)	CP188278.1	Ab2332v1		1	7
Belgium	CC2	ST2(2-2-2-2-2-2-2)	CP091336.1	AB233-VUB			5
China	CC2	ST2(2-2-2-2-2-2-2)	CP188275.1	Ab2342v1		1	3
China	CC2	ST2(2-2-2-2-2-2-2)	CP103413.1	AB2369		1	7
China	CC2	ST2(2-2-2-2-2-2-2)	CP092485.1	AB2877			5
Thailand	CC2	ST98(1-2-2-2-2-2-2)	CP091452.1	AB329			5

Country	CC	ST(Pasteur)	Accession	strain	Frameshifted	Truncated	Complete
unknown	CC2	ST2(2-2-2-2-2-2-2)	CP014291.1	AB34299			5
unknown	CC2	ST2(2-2-2-2-2-2-2)	CP104786.1	Ab-3556			2
unknown	CC2	ST2(2-2-2-2-2-2-2)	CP104907.1	Ab-3557			5
Belgium	CC2	ST2(2-2-2-2-2-2-2)	CP091371.1	AB36-VUB		1	6
China	CC2	ST2(2-2-2-2-2-2-2)	CP102831.1	AB3927		1	7
Belgium	CC2	ST2(2-2-2-2-2-2-2)	CP091370.1	AB39-VUB			3
Belgium	CC2	ST2(2-2-2-2-2-2-2)	CP091378.1	AB3-VUB			10
China	CC2	ST2(2-2-2-2-2-2-2)	CP107728.1	AB44			5
China	CC2	ST2(2-2-2-2-2-2-2)	CP095091.1	AB4451		1	7
China	CC2	ST2(2-2-2-2-2-2-2)	CP024613.1	Ab4568			5
China	CC2	ST2(2-2-2-2-2-2-2)	CP024612.1	Ab4653		1	9
China	CC2	ST2(2-2-2-2-2-2-2)	CP024611.1	Ab4977			6
China	CC2	ST2(2-2-2-2-2-2-2)	CP171791.1	AB50533		1	7
China	CC2	ST2(2-2-2-2-2-2-2)	CP159579.1	Ab6-Co-2			5
China	CC2	ST2(2-2-2-2-2-2-2)	CP054416.1	AB79		2	6
China	CC2	ST2(2-2-2-2-2-2-2)	CP030083.1	Aba			5
China	CC2	ST1555(2-2-181-2-2-2-2)	CP060732.1	abA1	1	1	5
Thailand	CC2	ST2(2-2-2-2-2-2-2)	CP042931.1	ABCR01			5
France	CC2	ST2(2-2-2-2-2-2-2)	CP136182.1	ABO21-A002		1	6
France	CC2	ST2(2-2-2-2-2-2-2)	CP136181.1	ABO21-A003		2	9
France	CC2	ST2(2-2-2-2-2-2-2)	CP136180.1	ABO21-A020		2	9
France	CC2	ST2(2-2-2-2-2-2-2)	CP136179.1	ABO21-A022		2	9
France	CC2	ST2(2-2-2-2-2-2-2)	CP136171.1	ABO21-A057		2	9
France	CC2	ST2(2-2-2-2-2-2-2)	CP136169.1	ABO21-A058		1	5
France	CC2	ST2(2-2-2-2-2-2-2)	CP136168.1	ABO21-A059			3
France	CC2	ST2(2-2-2-2-2-2-2)	CP136167.1	ABO21-A061		3	12
France	CC2	ST2(2-2-2-2-2-2-2)	CP136166.1	ABO21-A063		2	9
France	CC2	ST2(2-2-2-2-2-2-2)	CP136165.1	ABO21-A064		6	21
Pakistan	CC2	ST2(2-2-2-2-2-2-2)	CP024576.1	AbPK1			2
USA	CC2	ST2(2-2-2-2-2-2-2)	CP035051.1	ABUH763			3
USA	CC2	ST2(2-2-2-2-2-2-2)	CP035049.1	ABUH773			1
USA	CC2	ST2(2-2-2-2-2-2-2)	CP035045.1	ABUH793			3
USA	CC2	ST2(2-2-2-2-2-2-2)	CP035043.1	ABUH796			3
China	CC2	ST2(2-2-2-2-2-2-2)	CP053034.1	abYQ4	1	2	9
Malaysia	CC2	ST2(2-2-2-2-2-2-2)	CP007535.2	AC29			1
Thailand	CC2	ST2(2-2-2-2-2-2-2)	CP101653.1	Aci44			5
Germany	CC2	ST2(2-2-2-2-2-2-2)	CP086759.1	ACI713			4
Italy	CC2	ST2(2-2-2-2-2-2-2)	CP031380.1	ACICU			2
			CP031381.2	ACICU plasmid pACICU1b			2
USA	CC2	ST2(2-2-2-2-2-2-2)	CP018256.1	AF-673		2	5
Unknown	CC2	ST2(2-2-2-2-2-2-2)	CP027123.1	AR_0056		1	3
Unknown	CC2	ST2(2-2-2-2-2-2-2)	CP026707.1	AR_0056		1	3
			CP026705.1	AR_0056 plasmid tig00000058_pilon			4
Unknown	CC2	ST2(2-2-2-2-2-2-2)	CP027607.1	AR_0102			4
USA	CC2	ST2(2-2-2-2-2-2-2)	CP081144.1	ARLG_6295			2
USA	CC2	ST2(2-2-2-2-2-2-2)	CP081139.1	ARLG_6344			5
USA	CC2	ST2(2-2-2-2-2-2-2)	CP042841.1	ATCC BAA-1790			6
Australia	CC2	ST2(2-2-2-2-2-2-2)	CP024124.1	AYP-A2		1	9

Country	CC	ST(Pasteur)	Accession	strain	Frameshifted	Truncated	Complete
China	CC2	ST2(2-2-2-2-2-2-2)	CP079942.1	B10	1		7
South Korea	CC2	ST2(2-2-2-2-2-2-2)	CP142892.1	B20AB01			5
South Korea	CC2	ST2(2-2-2-2-2-2-2)	CP142101.1	B20AB06		1	5
South Korea	CC2	ST2(2-2-2-2-2-2-2)	CP142895.1	B20AB10			5
France	CC2	ST2(2-2-2-2-2-2-2)	CP059548.1	B9		1	3
UK	CC2	ST1550(2-226-2-2-2-2-2)	LT594095.1	BAL062			3
Viet Nam	CC2	ST2(2-2-2-2-2-2-2)	CP175804.1	BAL114	1	1	7
China	CC2	ST2(2-2-2-2-2-2-2)	CP003846.1	BJAB07104			2
			CP003907.1	BJAB07104 plasmid p2BJAB07104		2	1
China	CC2	ST2(2-2-2-2-2-2-2)	CP003849.1	BJAB0868			4
			CP003908.1	BJAB0868 plasmid p3BJAB0868		1	2
China	CC2	ST2(2-2-2-2-2-2-2)	CP091328.1	BM2333		1	6
South Korea	CC2	ST2(2-2-2-2-2-2-2)	CP142102.1	C20AB01			5
China	CC2	ST2(2-2-2-2-2-2-2)	CP032743.1	C25			6
South Korea	CC2	ST2(2-2-2-2-2-2-2)	CP020586.1	CBA7			6
USA	CC2	ST2(2-2-2-2-2-2-2)	CP061525.1	CFSAN093705			9
USA	CC2	ST2(2-2-2-2-2-2-2)	CP061523.1	CFSAN093706		1	9
USA	CC2	ST2(2-2-2-2-2-2-2)	CP061521.1	CFSAN093707		1	8
USA	CC2	ST2(2-2-2-2-2-2-2)	CP061519.1	CFSAN093708		1	9
USA	CC2	ST2(2-2-2-2-2-2-2)	CP061517.1	CFSAN093709		1	9
USA	CC2	ST2(2-2-2-2-2-2-2)	CP061514.1	CFSAN093710		1	9
Lebanon	CC2	ST2(2-2-2-2-2-2-2)	CP071763.1	Cl415			5
USA	CC2	ST2(2-2-2-2-2-2-2)	CP016295.1	CMC-CR-MDR-Ab4		2	7
USA	CC2	ST2(2-2-2-2-2-2-2)	CP016300.1	CMC-CR-MDR-Ab66		2	7
USA	CC2	ST2(2-2-2-2-2-2-2)	CP016298.1	CMC-MDR-Ab59		2	5
China	CC2	ST2(2-2-2-2-2-2-2)	CP184613.1	CQPMC-AB10			5
China	CC2	ST2(2-2-2-2-2-2-2)	CP184611.1	CQPMC-AB11			5
China	CC2	ST2(2-2-2-2-2-2-2)	CP184609.1	CQPMC-AB12		1	4
China	CC2	ST2(2-2-2-2-2-2-2)	CP184607.1	CQPMC-AB14			5
China	CC2	ST2(2-2-2-2-2-2-2)	CP184605.1	CQPMC-AB15			5
China	CC2	ST2(2-2-2-2-2-2-2)	CP184603.1	CQPMC-AB16			5
China	CC2	ST2(2-2-2-2-2-2-2)	CP184601.1	CQPMC-AB17		1	4
China	CC2	ST2(2-2-2-2-2-2-2)	CP184599.1	CQPMC-AB18			5
China	CC2	ST2(2-2-2-2-2-2-2)	CP184597.1	CQPMC-AB19		1	4
China	CC2	ST2(2-2-2-2-2-2-2)	CP184595.1	CQPMC-AB21			5
China	CC2	ST2(2-2-2-2-2-2-2)	CP184593.1	CQPMC-AB22			4
China	CC2	ST2(2-2-2-2-2-2-2)	CP184591.1	CQPMC-AB23			5
China	CC2	ST2(2-2-2-2-2-2-2)	CP184589.1	CQPMC-AB24			5
China	CC2	ST2(2-2-2-2-2-2-2)	CP184587.1	CQPMC-AB25			5
China	CC2	ST2(2-2-2-2-2-2-2)	CP184585.1	CQPMC-AB27			5
China	CC2	ST2(2-2-2-2-2-2-2)	CP184583.1	CQPMC-AB28			5
Thailand	CC2	ST2(2-2-2-2-2-2-2)	CP041148.1	CUVET-MIC596			4
Spain	CC2	ST2(2-2-2-2-2-2-2)	CP029569.1	DA33098			2
Germany	CC2	ST2(2-2-2-2-2-2-2)	CP087348.1	DB006			4
China	CC2	ST2(2-2-2-2-2-2-2)	CP075321.1	DD520		2	9
China	CC2	ST2(2-2-2-2-2-2-2)	CP077826.1	DETAB-E108		2	9
China	CC2	ST2(2-2-2-2-2-2-2)	CP077843.1	DETAB-E155			2
China	CC2	ST2(2-2-2-2-2-2-2)	CP077837.1	DETAB-E159			5

Country	CC	ST(Pasteur)	Accession	strain	Frameshifted	Truncated	Complete
China	CC2	ST2(2-2-2-2-2-2-2)	CP077828.1	DETAB-E351			5
China	CC2	ST2(2-2-2-2-2-2-2)	CP077830.1	DETAB-E51			2
China	CC2	ST2(2-2-2-2-2-2-2)	CP077846.1	DETAB-P24			5
China	CC2	ST2(2-2-2-2-2-2-2)	CP077832.1	DETAB-P43			2
China	CC2	ST2(2-2-2-2-2-2-2)	CP077840.1	DETAB-P90		1	6
China	CC2	ST2(2-2-2-2-2-2-2)	CP050916.1	DT-Ab003		2	9
China	CC2	ST2(2-2-2-2-2-2-2)	CP050914.1	DT-Ab007			7
China	CC2	ST2(2-2-2-2-2-2-2)	CP050911.1	DT-Ab020		2	9
China	CC2	ST2(2-2-2-2-2-2-2)	CP050907.1	DT-Ab022		2	9
China	CC2	ST2(2-2-2-2-2-2-2)	CP050904.1	DT-Ab057		1	4
South Korea	CC2	ST2(2-2-2-2-2-2-2)	CP017152.1	DU202		2	8
South Korea	CC2	ST2(2-2-2-2-2-2-2)	CP112859.1	EAB1		1	7
South Korea	CC2	ST2(2-2-2-2-2-2-2)	CP112860.1	EAB2		1	6
USA	CC2	ST2(2-2-2-2-2-2-2)	CP125225.1	EGA10			5
USA	CC2	ST2(2-2-2-2-2-2-2)	CP125223.1	EGA65			4
South Korea	CC2	ST2(2-2-2-2-2-2-2)	CP099969.1	F-1629		1	4
South Korea	CC2	ST2(2-2-2-2-2-2-2)	CP146229.1	F20AB03			4
Australia	CC2	ST2(2-2-2-2-2-2-2)	CP096575.1	F46	5	1	3
USA	CC2	ST2(2-2-2-2-2-2-2)	CP069851.1	FDAARGOS_1359		3	6
USA	CC2	ST2(2-2-2-2-2-2-2)	CP069840.1	FDAARGOS_1360			2
South Korea	CC2	ST2(2-2-2-2-2-2-2)	CP066237.1	G20AB007			5
South Korea	CC2	ST2(2-2-2-2-2-2-2)	CP066235.1	G20AB009			5
South Korea	CC2	ST2(2-2-2-2-2-2-2)	CP066232.1	G20AB010			5
South Korea	CC2	ST2(2-2-2-2-2-2-2)	CP066229.1	G20AB011			5
South Korea	CC2	ST2(2-2-2-2-2-2-2)	CP146231.1	G20AB08		1	6
USA	CC2	ST2(2-2-2-2-2-2-2)	CP174090.1	G636		1	7
Saudi Arabia	CC2	ST2(2-2-2-2-2-2-2)	CP121632.1	HAB11			2
China, Hongkong	CC2	ST2(2-2-2-2-2-2-2)	CP084733.1	HKU3			2
China, Hongkong	CC2	ST2(2-2-2-2-2-2-2)	CP084730.1	HKU4			2
China, Hongkong	CC2	ST2(2-2-2-2-2-2-2)	CP084727.1	HKU5			2
China, Hongkong	CC2	ST2(2-2-2-2-2-2-2)	CP084724.1	HKU6			2
China, Hongkong	CC2	ST2(2-2-2-2-2-2-2)	CP084721.1	HKU7			2
South Korea	CC2	ST2(2-2-2-2-2-2-2)	CP137140.1	HPA0438			2
South Korea	CC2	ST2(2-2-2-2-2-2-2)	CP137140.1	HPA0438			2
China	CC2	ST2(2-2-2-2-2-2-2)	CP018143.1	HRAB-85		1	6
China	CC2	ST2(2-2-2-2-2-2-2)	CP018143.1	HRAB-85		1	6
USA	CC2	ST2(2-2-2-2-2-2-2)	CP175646.1	HUMC1			10
USA	CC2	ST2(2-2-2-2-2-2-2)	CP169785.1	Hv652			2
Saudi Arabia	CC2	ST2(2-2-2-2-2-2-2)	CP121625.1	JAB144		1	8
Saudi Arabia	CC2	ST2(2-2-2-2-2-2-2)	CP121609.1	JAB186			4
Saudi Arabia	CC2	ST2(2-2-2-2-2-2-2)	CP121629.1	JAB270		1	4
Saudi Arabia	CC2	ST2(2-2-2-2-2-2-2)	CP121598.1	JAB77			5
South Korea	CC2	ST2(2-2-2-2-2-2-2)	CP020584.1	JBA13			4
Nepal	CC2	ST2(2-2-2-2-2-2-2)	AP031577.1	JUNP403		1	6
Nepal	CC2	ST2(2-2-2-2-2-2-2)	AP031578.1	JUNP405		1	6
Nepal	CC2	ST2(2-2-2-2-2-2-2)	AP031580.1	JUNP419			2
South Korea	CC2	ST2(2-2-2-2-2-2-2)	CP017642.1	KAB01		1	4
South Korea	CC2	ST2(2-2-2-2-2-2-2)	CP017644.1	KAB02			3

Country	CC	ST(Pasteur)	Accession	strain	Frameshifted	Truncated	Complete
South Korea	CC2	ST2(2-2-2-2-2-2-2)	CP017646.1	KAB03			5
South Korea	CC2	ST2(2-2-2-2-2-2-2)	CP017648.1	KAB04			5
South Korea	CC2	ST2(2-2-2-2-2-2-2)	CP017650.1	KAB05			3
South Korea	CC2	ST2(2-2-2-2-2-2-2)	CP017652.1	KAB06			3
South Korea	CC2	ST2(2-2-2-2-2-2-2)	CP017654.1	KAB07			4
South Korea	CC2	ST2(2-2-2-2-2-2-2)	CP017656.1	KAB08	1	1	2
South Korea	CC2	ST2(2-2-2-2-2-2-2)	CP013924.1	KBN10P02143		1	9
South Korea	CC2	ST2(2-2-2-2-2-2-2)	CP099989.1	KBN10P04593			2
South Korea	CC2	ST2(2-2-2-2-2-2-2)	CP100305.1	KBN10P05679			4
Greece	CC2	ST45(2-6-2-2-2-2-2)	CP031383.1	LUH 6011		3	8
Netherlands	CC2	ST2(2-2-2-2-2-2-2)	CP175648.1	LUH5537		2	12
			CP175650.1	LUH5537 plasmid pLUH5537-1			2
USA	CC2	ST195(2-2-2-2-2-1-2)	CP169828.1	Lv647			3
China	CC2	ST2716(2-2-2-2-615-2-2)	CP147665.1	LZFZ3604			2
Saudi Arabia	CC2	ST570(2-2-2-2-2-2-4)	CP121595.1	MAB17			7
Saudi Arabia	CC2	ST2(2-2-2-2-2-2-2)	CP121588.1	MAB9			6
China	CC2	ST2(2-2-2-2-2-2-2)	CP019114.1	MDR-CQ			5
China	CC2	ST2(2-2-2-2-2-2-2)	CP003500.1	MDR-TJ	1		4
USA	CC2	ST2(2-2-2-2-2-2-2)	CP031444.1	MDR-UNC			4
			CP031445.1	MDR-UNC plasmid unnamed1		2	2
China	CC2	ST2(2-2-2-2-2-2-2)	CP001937.2	MDR-ZJ06		1	5
Australia	CC2	ST2(2-2-2-2-2-2-2)	CP054302.1	MS14413		1	5
Thailand	CC2	ST2(2-2-2-2-2-2-2)	CP160307.1	MTC0608			5
Thailand	CC2	ST2(2-2-2-2-2-2-2)	CP162568.1	MTC0609			5
Thailand	CC2	ST2(2-2-2-2-2-2-2)	CP160306.1	MTC0617			4
Thailand	CC2	ST2(2-2-2-2-2-2-2)	CP162561.1	MTC0620			5
Thailand	CC2	ST2(2-2-2-2-2-2-2)	CP162558.1	MTC0629			3
Thailand	CC2	ST2(2-2-2-2-2-2-2)	CP162554.1	MTC1120			5
USA	CC2	ST2(2-2-2-2-2-2-2)	CP096894.1	Mu1956		1	6
USA	CC2	ST2(2-2-2-2-2-2-2)	CP096818.1	Mu1984			7
Canada	CC2	ST2(2-2-2-2-2-2-2)	CP043417.1	N13-03449		2	7
China	CC2	ST2(2-2-2-2-2-2-2)	CP159528.1	NAB01B			4
China	CC2	ST2(2-2-2-2-2-2-2)	CP159519.1	NAB01B-R1			4
China	CC2	ST2(2-2-2-2-2-2-2)	CP159626.1	NAB01B-R1			4
China	CC2	ST2(2-2-2-2-2-2-2)	CP159527.1	NAB01B-R10			4
China	CC2	ST2(2-2-2-2-2-2-2)	CP159520.1	NAB01B-R3			4
China	CC2	ST2(2-2-2-2-2-2-2)	CP159522.1	NAB01B-R4			4
China	CC2	ST2(2-2-2-2-2-2-2)	CP159521.1	NAB01B-R5			4
China	CC2	ST2(2-2-2-2-2-2-2)	CP159523.1	NAB01B-R6			4
China	CC2	ST2(2-2-2-2-2-2-2)	CP159525.1	NAB01B-R7			4
China	CC2	ST2(2-2-2-2-2-2-2)	CP159524.1	NAB01B-R8			4
China	CC2	ST2(2-2-2-2-2-2-2)	CP159526.1	NAB01B-R9			4
China	CC2	ST2(2-2-2-2-2-2-2)	CP158366.1	NAB02B			4
China	CC2	ST2(2-2-2-2-2-2-2)	CP159516.1	NAB03B			4
China	CC2	ST2(2-2-2-2-2-2-2)	CP159517.1	NAB04B			4
China	CC2	ST2(2-2-2-2-2-2-2)	CP159624.1	NAB05B			4
China	CC2	ST2(2-2-2-2-2-2-2)	CP159625.1	NAB06B			4
China	CC2	ST2(2-2-2-2-2-2-2)	CP159518.1	NAB07B			4

Country	CC	ST(Pasteur)	Accession	strain	Frameshifted	Truncated	Complete
South Korea	CC2	ST2(2-2-2-2-2-2-2)	CP099784.1	NCCP 15989	3	1	2
South Korea	CC2	N8(2-2-2-2-2-2#-2)	CP099786.1	NCCP 15992	3	2	5
South Korea	CC2	ST2(2-2-2-2-2-2-2)	CP099788.1	NCCP 15995	4	2	2
South Korea	CC2	ST2(2-2-2-2-2-2-2)	CP099790.1	NCCP 15996			5
South Korea	CC2	ST2(2-2-2-2-2-2-2)	CP099793.1	NCCP 16006	2	1	4
South Korea	CC2	ST2(2-2-2-2-2-2-2)	CP091465.1	NCCP 16007		1	6
South Korea	CC2	ST2(2-2-2-2-2-2-2)	CP099795.1	NCCP 16011		1	6
Japan	CC2	ST2(2-2-2-2-2-2-2)	AP013357.1	NCGM 237			3
USA	CC2	ST2(2-2-2-2-2-2-2)	CP175653.1	NIH1			5
Cambodia	CC2	ST571(2-2-2-2-104-2-4)	AP024415.1	NIPH17_0019	5		
unknown	CC2	ST2(2-2-2-2-2-2-2)	CP106988.1	NY13623			6
China	CC2	ST2(2-2-2-2-2-2-2)	CP094283.1	NY5301		1	8
Germany	CC2	ST2(2-2-2-2-2-2-2)	CP087321.1	OC043			4
Japan	CC2	ST2(2-2-2-2-2-2-2)	AP025531.1	OCU-Ac19			5
Japan	CC2	ST2(2-2-2-2-2-2-2)	AP025535.1	OCU-Ac20			2
USA	CC2	ST2(2-2-2-2-2-2-2)	CP015483.1	ORAB01		2	7
USA	CC2	ST2(2-2-2-2-2-2-2)	CP040425.1	PB364			4
			CP040426.1	PB364 plasmid pPB364_1			2
India	CC2	ST2(2-2-2-2-2-2-2)	CP050410.1	PM1912235			5
India	CC2	ST2(2-2-2-2-2-2-2)	CP050412.1	PM192696			2
Paraguay	CC2	ST2(2-2-2-2-2-2-2)	CP179709.1	PR1			5
Paraguay	CC2	ST2(2-2-2-2-2-2-2)	CP179707.1	PR2			7
Paraguay	CC2	ST2(2-2-2-2-2-2-2)	CP179705.1	PR3			7
Paraguay	CC2	ST2(2-2-2-2-2-2-2)	CP179702.1	PR4			6
Paraguay	CC2	ST2(2-2-2-2-2-2-2)	CP179699.1	PR5			7
Paraguay	CC2	ST2(2-2-2-2-2-2-2)	CP179696.1	PR6			7
Paraguay	CC2	ST2(2-2-2-2-2-2-2)	CP179692.1	PR8			7
China	CC2	ST2(2-2-2-2-2-2-2)	CP097875.1	R4-1	4	2	5
China	CC2	ST2(2-2-2-2-2-2-2)	CP097878.1	R6-1	4	2	4
Saudi Arabia	CC2	ST2(2-2-2-2-2-2-2)	CP121586.1	RAB11			2
Saudi Arabia	CC2	ST2(2-2-2-2-2-2-2)	CP121583.1	RAB14			5
Saudi Arabia	CC2	ST1579(2-229-2-2-2-2-2)	CP121579.1	RAB53			2
Saudi Arabia	CC2	ST2(2-2-2-2-2-2-2)	CP121577.1	RAB55			2
Saudi Arabia	CC2	ST1580(2-230-2-2-2-2-2)	CP121567.1	RAB73		1	5
Saudi Arabia	CC2	ST1579(2-229-2-2-2-2-2)	CP121557.1	RAB9			2
Saudi Arabia	CC2	ST570(2-2-2-2-2-2-4)	CP121563.1	RAB94		1	8
Saudi Arabia	CC2	ST1579(2-229-2-2-2-2-2)	CP121560.1	RAB97			2
Nigeria	CC2	ST2(2-2-2-2-2-2-2)	CP178386.1	RP15			6
USA	CC2	ST2(2-2-2-2-2-2-2)	CP169848.1	Rp436			2
USA	CC2	ST2(2-2-2-2-2-2-2)	CP169852.1	Rp654			3
South Korea	CC2	ST187(2-2-2-2-2-2-43)	CP020579.1	SAA14			4
Russia	CC2	ST2(2-2-2-2-2-2-2)	CP157866.1	SCCH68:Ab991128		2	5
Singapore	CC2	ST2(2-2-2-2-2-2-2)	CP161992.1	SIMBA034			4
South Korea	CC2	ST2(2-2-2-2-2-2-2)	CP025266.1	SMC_Paed_Ab_BL01		1	10
South Korea	CC2	ST2(2-2-2-2-2-2-2)	CP137069.1	SNUBHAB0062			7
South Korea	CC2	ST2(2-2-2-2-2-2-2)	CP137073.1	SNUBHAB0197			2
South Korea	CC2	ST2(2-2-2-2-2-2-2)	CP137071.1	SNUBHAB0220			8
South Korea	CC2	ST2(2-2-2-2-2-2-2)	CP137061.1	SNUBHAB0274			5

Country	CC	ST(Pasteur)	Accession	strain	Frameshifted	Truncated	Complete
South Korea	CC2	ST2(2-2-2-2-2-2-2)	CP137066.1	SNUBHAB0277			3
South Korea	CC2	ST2(2-2-2-2-2-2-2)	CP137059.1	SNUBHAB0291			5
South Korea	CC2	ST2(2-2-2-2-2-2-2)	CP137064.1	SNUBHAB0292			4
China	CC2	ST2(2-2-2-2-2-2-2)	CP152383.1	SRM1			8
China	CC2	ST2(2-2-2-2-2-2-2)	CP144238.1	SRM21			6
China	CC2	ST2(2-2-2-2-2-2-2)	CP144240.1	SRM25		1	6
South Korea	CC2	ST2(2-2-2-2-2-2-2)	CP020578.1	SSA12			4
South Korea	CC2	ST2(2-2-2-2-2-2-2)	CP020591.1	SSA6		2	6
South Korea	CC2	ST2(2-2-2-2-2-2-2)	CP020581.1	SSMA17			4
Nepal	CC2	ST2(2-2-2-2-2-2-2)	CP048102.1	SSRI1		1	7
Turkey	CC2	ST2(2-2-2-2-2-2-2)	CP171394.1	T3			1
China, Taiwan	CC2	ST2(2-2-2-2-2-2-2)	CP002522.2	TCDC-AB0715			7
USA	CC2	ST2(2-2-2-2-2-2-2)	CP039993.1	TG22182		2	5
USA	CC2	ST2(2-2-2-2-2-2-2)	CP039520.1	TG22627		1	3
USA	CC2	ST2(2-2-2-2-2-2-2)	CP039518.1	TG22653			3
USA	CC2	ST570(2-2-2-2-2-2-4)	CP056784.2	TP1			7
USA	CC2	ST570(2-2-2-2-2-2-4)	CP060011.1	TP2		1	8
USA	CC2	ST570(2-2-2-2-2-2-4)	CP060013.1	TP3		1	8
China	CC2	ST2(2-2-2-2-2-2-2)	CP155637.1	TY918			6
China, Taiwan	CC2	ST2(2-2-2-2-2-2-2)	CP003856.1	TYTH-1		1	3
USA	CC2	ST2(2-2-2-2-2-2-2)	CP175656.1	UMB001			6
USA	CC2	ST2(2-2-2-2-2-2-2)	CP190383.1	Up644			2
USA	CC2	ST2(2-2-2-2-2-2-2)	CP190387.1	Up645		1	4
South Korea	CC2	ST2(2-2-2-2-2-2-2)	CP020592.1	USA2			3
India	CC2	ST2(2-2-2-2-2-2-2)	CP050400.1	VB11737			2
India	CC2	N1(2-307-2-2-2-2-2)	CP040047.1	VB1190	1		
			CP040048.1	VB1190 plasmid unnamed1	1	1	1
India	CC2	ST2(2-2-2-2-2-2-2)	CP051474.1	VB2107			5
India	CC2	ST2(2-2-2-2-2-2-2)	CP050526.1	VB2139			2
India	CC2	ST2(2-2-2-2-2-2-2)	CP050401.1	VB2181			6
India	CC2	ST2(2-2-2-2-2-2-2)	CP050421.1	VB2200			2
India	CC2	ST2(2-2-2-2-2-2-2)	CP035672.1	VB23193	5	1	4
India	CC2	N2(2-307#-2-2-2-2-460)	CP035930.1	VB31459	4	1	2
			CP035931.1	VB31459 plasmid unnamed1	1		1
India	CC2	ST2(2-2-2-2-2-2-2)	CP040084.1	VB33071		1	3
			CP040085.1	VB33071 plasmid unnamed1			1
India	CC2	ST2(2-2-2-2-2-2-2)	CP040087.1	VB35575	2	1	6
India	CC2	ST2(2-2-2-2-2-2-2)	CP050388.1	VB473		1	3
India	CC2	ST2(2-2-2-2-2-2-2)	CP050523.1	VB7036			4
India	CC2	ST2(2-2-2-2-2-2-2)	CP050390.1	VB723			5
China	CC2	ST2(2-2-2-2-2-2-2)	CP163039.1	W155			7
China	CC2	ST2(2-2-2-2-2-2-2)	CP026750.2	WCHAB005133		1	9
			CP026749.2	WCHAB005133 plasmid pOXA58_005133			2
Australia	CC2	ST2(2-2-2-2-2-2-2)	CP191379.1	WM99a		1	5
China, Taiwan	CC2	ST724(2-2-2-2-2-2-5)	CP074695.1	X4-107			9
China, Taiwan	CC2	ST724(2-2-2-2-2-2-5)	CP074698.1	X4-136			9
China, Taiwan	CC2	ST724(2-2-2-2-2-2-5)	CP076736.1	X4-201			9
China, Taiwan	CC2	ST724(2-2-2-2-2-2-5)	CP064203.1	X4-300			9

Country	CC	ST(Pasteur)	Accession	strain	Frameshifted	Truncated	Complete
China, Taiwan	CC2	ST724(2-2-2-2-2-2-5)	CP076739.1	X4-584			9
China, Taiwan	CC2	ST724(2-2-2-2-2-2-5)	CP064194.1	X4-65			9
China, Taiwan	CC2	ST724(2-2-2-2-2-2-5)	CP076742.1	X4-705			9
China	CC2	ST2(2-2-2-2-2-2-2)	CP018421.1	XDR-BJ83		1	4
China	CC2	ST2(2-2-2-2-2-2-2)	CP134599.1	XH1015		2	9
China	CC2	ST2(2-2-2-2-2-2-2)	CP134597.1	XH1016		1	6
China	CC2	ST2(2-2-2-2-2-2-2)	CP134596.1	XH1017		1	8
China	CC2	ST2(2-2-2-2-2-2-2)	CP134595.1	XH1018		2	8
China	CC2	ST2(2-2-2-2-2-2-2)	CP134594.1	XH1019		1	5
China	CC2	ST2(2-2-2-2-2-2-2)	CP134591.1	XH1023			8
China	CC2	ST2(2-2-2-2-2-2-2)	CP134580.1	XH1033		2	8
China	CC2	ST2(2-2-2-2-2-2-2)	CP134574.1	XH1035		1	4
China	CC2	ST2(2-2-2-2-2-2-2)	CP134571.1	XH1036		1	5
China	CC2	ST2(2-2-2-2-2-2-2)	CP134570.1	XH1037		2	8
China	CC2	ST2(2-2-2-2-2-2-2)	CP134565.1	XH1039			6
China	CC2	ST2(2-2-2-2-2-2-2)	CP134564.1	XH1040		1	4
China	CC2	ST2(2-2-2-2-2-2-2)	CP134560.1	XH1042		1	7
China	CC2	ST2(2-2-2-2-2-2-2)	CP134558.1	XH1043		1	4
China	CC2	ST104(2-2-2-2-2-2-14)	CP134553.1	XH1045		1	5
China	CC2	ST2(2-2-2-2-2-2-2)	CP134550.1	XH1048		2	8
China	CC2	ST2(2-2-2-2-2-2-2)	CP134549.1	XH1050			7
China	CC2	ST2(2-2-2-2-2-2-2)	CP134547.1	XH1052		1	6
China	CC2	ST2(2-2-2-2-2-2-2)	CP134544.1	XH1054		2	8
China	CC2	ST2(2-2-2-2-2-2-2)	CP134540.1	XH1055			6
China	CC2	ST2(2-2-2-2-2-2-2)	CP096892.1	XH1820			5
China	CC2	ST2(2-2-2-2-2-2-2)	CP021326.1	XH386		2	8
China	CC2	ST2(2-2-2-2-2-2-2)	CP010779.1	XH386		2	8
China	CC2	ST2(2-2-2-2-2-2-2)	CP019217.1	XH731			2
China	CC2	ST2(2-2-2-2-2-2-2)	CP021321.1	XH731			2
China	CC2	ST2(2-2-2-2-2-2-2)	CP014541.1	XH856		2	8
China	CC2	ST2(2-2-2-2-2-2-2)	CP014539.1	XH859			7
China	CC2	ST2(2-2-2-2-2-2-2)	CP014538.1	XH860			5
China	CC2	ST922(2-2-2-75-2-2-2)	CP023140.1	XH906			5
China	CC2	ST2(2-2-2-2-2-2-2)	CP060285.1	XYAB2018			5
China	CC2	ST2(2-2-2-2-2-2-2)	CP144247.1	YFY21		1	7
China	CC2	ST2(2-2-2-2-2-2-2)	CP144249.1	YFY24			5
China	CC2	ST2(2-2-2-2-2-2-2)	CP144252.1	YFY27		1	6
China	CC2	ST2(2-2-2-2-2-2-2)	CP144255.1	YFY3		1	6
Korea	CC2	ST2(2-2-2-2-2-2-2)	CP014215.1	YU-R612			5
china	CC2	ST2(2-2-2-2-2-2-2)	CP104908.1	YZM-0314			2
china	CC2	ST2(2-2-2-2-2-2-2)	CP104912.1	YZM-0406			5
China	CC2	ST2(2-2-2-2-2-2-2)	CP109836.1	Z198			2
Mexico	CC1	ST1(1-1-1-1-5-1-1)	CP023029.1	9102			
UK	CC1	ST1(1-1-1-1-5-1-1)	CP041035.1	11W359501			1
UK	CC1	ST1(1-1-1-1-5-1-1)	CP010781.1	A1			1
Netherlands	CC1	ST1(1-1-1-1-5-1-1)	CP178354.2	A297/RUH875		1	3
Greece	CC1	ST1(1-1-1-1-5-1-1)	CP024418.1	A388		1	5
			CP024419.1	A388 plasmid pA388			3

Country	CC	ST(Pasteur)	Accession	strain	Frameshifted	Truncated	Complete
Australia	CC1	ST1(1-1-1-1-5-1-1)	CP021782.1	A85		1	3
USA	CC1	ST1(1-1-1-1-5-1-1)	CP001182.2	AB0057		1	3
Belgium	CC1	ST1(1-1-1-1-5-1-1)	CP091367.1	AB169-VUB		1	3
Belgium	CC1	ST1(1-1-1-1-5-1-1)	CP091340.1	AB227-VUB			
Belgium	CC1	ST1(1-1-1-1-5-1-1)	CP091339.1	AB229-VUB			
Belgium	CC1	ST1(1-1-1-1-5-1-1)	CP091337.1	AB232-VUB			
USA	CC1	ST1(1-1-1-1-5-1-1)	CP001172.2	AB307-0294			
China, Taiwan	CC1	ST1(1-1-1-1-5-1-1)	CP119232.1	AB322	1	2	6
USA	CC1	ST1(1-1-1-1-5-1-1)	CP113078.1	AB5075			
USA	CC1	ST1(1-1-1-1-5-1-1)	CP144559.1	AB5075			
UK	CC1	ST1(1-1-1-1-5-1-1)	CP144563.1	AB5075 isolate grey variant			
unknown	CC1	ST1(1-1-1-1-5-1-1)	CP113080.1	AB5075-T			
USA	CC1	ST1(1-1-1-1-5-1-1)	CP008706.1	AB5075-UW			
Belgium	CC1	ST1(1-1-1-1-5-1-1)	CP070362.2	AB5075-VUB			
Belgium	CC1	ST1(1-1-1-1-5-1-1)	CP070358.2	AB5075-VUB-itrA::ISAba13			
Australia	CC1	ST1(1-1-1-1-5-1-1)	CP114381.1	AB6870155		1	3
Unknown	CC1	ST1(1-1-1-1-5-1-1)	CP027528.1	AR_0083		1	4
Canada	CC1	ST1(1-1-1-1-5-1-1)	CP058625.1	ATCC BAA1605		1	3
Lebanon	CC1	ST1(1-1-1-1-5-1-1)	CP049240.1	Ax270			
France	CC1	ST1(1-1-1-1-5-1-1)	CU459141.1	AYE		1	3
South Korea	CC1	ST1(1-1-1-1-5-1-1)	CP143262.1	C20AB05			3
Canada	CC1	ST1(1-1-1-1-5-1-1)	CP116680.1	Canada BC-5			1
Australia	CC1	ST1(1-1-1-1-5-1-1)	CP156044.1	D13		1	2
Australia	CC1	ST81(1-1-1-1-5-1-2)	CP012952.1	D36			1
			CP012956.1	D36 plasmid pD36-4			3
Australia	CC1	ST1(1-1-1-1-5-1-1)	CP156041.1	D78			1
			CP156043.1	D78 plasmid pD78-1		2	3
Germany	CC1	ST1(1-1-1-1-5-1-1)	CP030106.1	DA33382		1	3
			CP030109.1	DA33382 plasmid pDA33382-85			1
Lebanon	CC1	ST1(1-1-1-1-5-1-1)	CP049314.1	Ex003			
USA	CC1	ST1(1-1-1-1-5-1-1)	CP066016.1	FDAARGOS_1036		1	3
Australia	CC1	ST1(1-1-1-1-5-1-1)	CP175642.1	G7		1	3
France	CC1	ST1(1-1-1-1-5-1-1)	CP169788.1	Hv766			
Nepal	CC1	ST1(1-1-1-1-5-1-1)	AP031576.1	JUNP402		1	4
Nepal	CC1	ST1(1-1-1-1-5-1-1)	AP031588.1	JUNP712		1	4
USA	CC1	ST1(1-1-1-1-5-1-1)	CP080452.1	MRSN 56		1	2
USA	CC1	ST1(1-1-1-1-5-1-1)	CP090606.1	MRSN 56-T2	1	57	57
USA	CC1	ST1(1-1-1-1-5-1-1)	CP090607.1	MRSN 58		76	153
USA	CC1	ST1(1-1-1-1-5-1-1)	CP091172.1	MRSN57		8	15
Afghanistan	CC1	ST1(1-1-1-1-5-1-1)	CP130627.2	MRSN571146		1	2
Afghanistan	CC1	ST1(1-1-1-1-5-1-1)	CP130628.2	MRSN576822		1	2
Viet Nam	CC1	ST1(1-1-1-1-5-1-1)	CP154372.1	NCSR_106			1
UK	CC1	ST1(1-1-1-1-5-1-1)	LS483472.1	NCTC13421		1	3
Germany	CC1	ST1(1-1-1-1-5-1-1)	CP139831.1	Nord12-3		1	2
			CP139835.1	Nord12-3 plasmid pNord12_5		1	2
Germany	CC1	ST1(1-1-1-1-5-1-1)	CP087317.1	OC064			7
			CP087319.1	OC064 plasmid p1OC064			4
			CP087318.1	OC064 plasmid p2OC064	1	1	3

Country	CC	ST(Pasteur)	Accession	strain	Frameshifted	Truncated	Complete
Germany	CC1	ST1(1-1-1-1-5-1-1)	CP087304.1	OC081			1
Greece	CC1	ST1(1-1-1-1-5-1-1)	CP169833.1	Rp376			2
Singapore	CC1	ST1(1-1-1-1-5-1-1)	CP162145.1	SIMBA089		1	2
Chile	CC1	ST1(1-1-1-1-5-1-1)	CP076821.1	UC22850			1
			CP076822.1	UC22850 plasmid p1UC22850			2
South Korea	CC1	ST1(1-1-1-1-5-1-1)	CP020595.1	USA15			
India	CC1	ST1(1-1-1-1-5-1-1)	CP050403.1	VB2486			
China	CC1	ST20(3-1-1-1-5-1-1)	CP027246.2	WCHAB005078			3
Australia	CC1	ST1(1-1-1-1-5-1-1)	CP116387.1	WM98		1	3
China	CC1	ST1(1-1-1-1-5-1-1)	CP134552.1	XH1047			
Pakistan	CC25	ST307(3-3-2-4-7-2-30)	CP142393.1	38			
Mexico	CC25	ST25(3-3-2-4-7-2-4)	CP022283.1	7804			
France	CC25	ST25(3-3-2-4-7-2-4)	CP077801.1	40288			
France	CC25	ST25(3-3-2-4-7-2-4)	CP129245.1	13A297n			
unknown	CC25	ST25(3-3-2-4-7-2-4)	CP188770.1	15A-STR			
France	CC25	ST25(3-3-2-4-7-2-4)	CP151688.1	40288-CIRI			
Germany	CC25	ST25(3-3-2-4-7-2-4)	CP181857.1	A26326			
			CP181858.1	A26326 plasmid p1_A26326			3
Germany	CC25	ST25(3-3-2-4-7-2-4)	CP181854.1	A26329			
			CP181855.1	A26329 plasmid p1_A26329	1		2
Unknown	CC25	ST25(3-3-2-4-7-2-4)	CP027530.1	AR_0088			
Lebanon	CC25	ST25(3-3-2-4-7-2-4)	CP098521.1	CI107			
Australia	CC25	ST25(3-3-2-4-7-2-4)	CP048849.1	D4			2
Australia	CC25	ST25(3-3-2-4-7-2-4)	CP048131.1	D46			
			CP048135.1	D46 plasmid pD46-4			4
France	CC25	ST25(3-3-2-4-7-2-4)	CP151719.1	F14-11			
France	CC25	ST25(3-3-2-4-7-2-4)	CP151717.1	F15-01A			
France	CC25	ST25(3-3-2-4-7-2-4)	CP151715.1	F15-01B			
France	CC25	ST25(3-3-2-4-7-2-4)	CP151713.1	F15-02			
France	CC25	ST25(3-3-2-4-7-2-4)	CP151711.1	F15-06			
France	CC25	ST25(3-3-2-4-7-2-4)	CP151709.1	F16-05			
France	CC25	ST25(3-3-2-4-7-2-4)	CP151707.1	F18-02			
France	CC25	ST25(3-3-2-4-7-2-4)	CP151705.1	F19-02			
France	CC25	ST25(3-3-2-4-7-2-4)	CP151704.1	F19-10-2			
France	CC25	ST25(3-3-2-4-7-2-4)	CP151702.1	F19-10-4			
France	CC25	ST25(3-3-2-4-7-2-4)	CP151700.1	F19-10-8			
France	CC25	ST25(3-3-2-4-7-2-4)	CP151699.1	F19-10-9			
France	CC25	ST25(3-3-2-4-7-2-4)	CP151694.1	F19-10-CH3			
France	CC25	ST25(3-3-2-4-7-2-4)	CP151692.1	F19-10-CH6			
France	CC25	ST25(3-3-2-4-7-2-4)	CP151690.1	F19-10-CH8			
France	CC25	ST25(3-3-2-4-7-2-4)	CP151696.1	F19-10-MH12			
France	CC25	ST25(3-3-2-4-7-2-4)	CP151697.1	F19-10-MH6			
Belgium	CC25	ST25(3-3-2-4-7-2-4)	CP169766.1	Hv373			
unknown	CC25	N14(3-3-2-4$^{\#}$-7-2-4)	CP169776.1	Hv455			
			CP169777.1	Hv455 plasmid unnamed1			1
South Korea	CC25	ST25(3-3-2-4-7-2-4)	CP020597.1	HWBA8			
			CP020596.1	HWBA8 plasmid pHWBA8_1			3
Nepal	CC25	N15(3-3-2-4-7-2-N)	AP031586.1	JUNP586			

Country	CC	ST(Pasteur)	Accession	strain	Frameshifted	Truncated	Complete
			AP031587.1	JUNP586 plasmid pJUNP586			2
Bolivia	CC25	ST25(3-3-2-4-7-2-4)	CP169809.1	Lv347			
Germany	CC25	ST25(3-3-2-4-7-2-4)	CP091596.1	Nord4-2			2
India	CC25	ST25(3-3-2-4-7-2-4)	CP040259.1	P7774			
			CP040260.1	P7774 plasmid unnamed1			2
Argentina	CC25	ST25(3-3-2-4-7-2-4)	CP169872.1	Up377			
			CP169873.1	Up377 plasmid unnamed1			1
India	CC25	ST25(3-3-2-4-7-2-4)	CP050385.1	VB82			
			CP050386.1	VB82 plasmid pVB82_1			3
Mexico	CC23	ST10(1-3-2-1-4-4-4)	CP023022.1	10324			
Canada	CC23	ST10(1-3-2-1-4-4-4)	CP012006.1	Ab04-mff			
			CP012007.1	Ab04-mff plasmid pAB04-1			3
Belgium	CC23	ST10(1-3-2-1-4-4-4)	CP091360.1	AB179-VUB			
China	CC23	ST23(1-3-10-1-4-4-4)	CP048827.1	ABF9692			
China	CC23	ST23(1-3-10-1-4-4-4)	CP003847.1	BJAB0715			
India	CC23	ST10(1-3-2-1-4-4-4)	CP038500.1	CIAT758			
			CP038501.1	CIAT758 plasmid unnamed1		1	
			CP038502.1	CIAT758 plasmid unnamed2	2		2
South Korea	CC23	ST10(1-3-2-1-4-4-4)	CP173428.1	CRAB_1			
unknown	CC23	ST10(1-3-2-1-4-4-4)	CP169805.1	HvAb04			
			CP169806.1	HvAb04 plasmid unnamed1			3
Nepal	CC23	ST10(1-3-2-1-4-4-4)	AP031582.1	JUNP499			2
USA	CC23	ST10(1-3-2-1-4-4-4)	CP018677.1	LAC4			
USA	CC23	ST10(1-3-2-1-4-4-4)	CP007712.1	LAC-4			
India	CC23	ST10(1-3-2-1-4-4-4)	CP050415.1	PM193665			
			CP050416.1	PM193665 plasmid pPM193665_1		1	1
India	CC23	ST10(1-3-2-1-4-4-4)	CP050425.1	PM194188			
			CP050426.1	PM194188 plasmid pPM194122_1		1	1
India	CC23	ST10(1-3-2-1-4-4-4)	CP050432.1	PM194229			
			CP050433.1	PM194229 plasmid pPM194229_1		1	1
UNKNOW	CC23	ST10(1-3-2-1-4-4-4)	CP144907.1	PUMA0247			
UNKNOW	CC23	ST10(1-3-2-1-4-4-4)	CP144757.1	PUMA0248			1
Singapore	CC23	ST10(1-3-2-1-4-4-4)	CP161986.1	SIMBA003			
Singapore	CC23	ST10(1-3-2-1-4-4-4)	CP161989.1	SIMBA028			
Singapore	CC23	ST575(1-3-2-1-4-4-92)	CP161995.1	SIMBA035			
South Korea	CC23	ST10(1-3-2-1-4-4-4)	CP137075.1	SNUBHAB0006			
India	CC23	ST1512(1-3-10-1-4-1-4)	CP040053.1	VB35179			
			CP040054.1	VB35179 plasmid unnamed1	1		1
India	CC23	ST575(1-3-2-1-4-4-92)	CP040056.1	VB35435			
			CP040057.1	VB35435 plasmid unnamed1	1		1
South Korea	CC23	ST10(1-3-2-1-4-4-4)	CP020598.1	WKA02			
China	CC23	ST23(1-3-10-1-4-4-4)	CP134562.1	XH1041			
China	CC23	ST10(1-3-2-1-4-4-4)	CP134548.1	XH1051			
China	CC23	ST256(1-3-10-1-2-4-4)	CP045645.1	XH1056			2
China	CC23	ST10(1-3-2-1-4-4-4)	CP128372.1	XH2146			
China	CC23	ST23(1-3-10-1-4-4-4)	CP014528.1	XH858			
Mexico	CC79	ST422(26-72-2-2-29-4-5)	CP015364.1	3207			
Mexico	CC79	ST422(26-72-2-2-29-4-5)	CP033243.1	7835			

Country	CC	ST(Pasteur)	Accession	strain	Frameshifted	Truncated	Complete
Mexico	CC79	ST422(26-72-2-2-29-4-5)	CP023020.1	9201			3
USA	CC79	ST422(26-72-2-2-29-4-5)	CP104342.1	2021CK-01300			3
USA	CC79	ST422(26-72-2-2-29-4-5)	CP104347.1	2021CK-01332			3
USA	CC79	ST422(26-72-2-2-29-4-5)	CP104350.1	2021CK-01333			3
USA	CC79	ST422(26-72-2-2-29-4-5)	CP104351.1	2021CK-01335			3
USA	CC79	ST422(26-72-2-2-29-4-5)	CP104448.1	2021CK-01407			3
USA	CC79	ST422(26-72-2-2-29-4-5)	CP104335.1	2021CK-01408			3
USA	CC79	ST422(26-72-2-2-29-4-5)	CP104340.1	2021CK-01409			3
Mexico	CC79	ST156(26-2-2-2-29-4-4)	CP026338.1	810CP			1
Canada	CC79	ST79(26-2-2-2-29-4-5)	CP009257.1	AB030			2
Spain	CC79	ST79(26-2-2-2-29-4-5)	CP009534.1	AbH12O-A2			1
Mexico	CC79	ST79(26-2-2-2-29-4-5)	CP018254.1	AF-401			1
Argentina	CC79	ST79(26-2-2-2-29-4-5)	CP169298.1	AMA205			5
Unknown	CC79	ST79(26-2-2-2-29-4-5)	CP026711.1	AR_0063			1
Unknown	CC79	ST79(26-2-2-2-29-4-5)	CP027611.1	AR_0101			1
USA	CC79	ST79(26-2-2-2-29-4-5)	CP190359.1	Hv637			1
USA	CC79	ST79(26-2-2-2-29-4-5)	CP169819.1	Lv419			1
Brazil	CC79	ST79(26-2-2-2-29-4-5)	CP033869.1	MRSN15313			1
Paraguay	CC79	ST79(26-2-2-2-29-4-5)	CP179694.1	PR7			1
unknown	CC79	ST298(1-2-2-2-29-4-5)	CP169839.1	Rp426			2
unknown	CC79	ST79(26-2-2-2-29-4-5)	CP169842.1	Rp428			
			CP169845.1	Rp428 plasmid unnamed3		1	2
Chile	CC79	ST79(26-2-2-2-29-4-5)	CP076812.1	UC23022			1
			CP076813.1	UC23022 plasmid p1UC23022			2
unknown	CC374	ST437(3-2-2-2-30-4-28)	CP099855.1	17978R			
unknown	CC374	ST437(3-2-2-2-30-4-28)	CP099856.1	17978S			
France	CC374	ST437(3-2-2-2-30-4-28)	CP079931.1	17978UN			
China	CC374	ST77(3-2-2-2-3-4-28)	CP034092.1	A52			
unknown	CC374	ST437(3-2-2-2-30-4-28)	CP019034.1	AB042			
unknown	CC374	ST437(3-2-2-2-30-4-28)	CP043910.1	AB043			
India	CC374	N13(3-2-2-2-44-1-4)	CP185768.1	AB05			
Canada	CC374	ST825(3-2-2-2-7-4-4)	CP045428.1	AbCAN2			
USA	CC374	ST437(3-2-2-2-30-4-28)	CP053098.1	ATCC 17978			
France	CC374	ST437(3-2-2-2-30-4-28)	CP091335.1	ATCC17978-VUB			
Brazil	CC374	N3(3#-2-2-2-3-4-4)	CP101889.1	CCBH31258			
			CP101888.1	CCBH31258 plasmid pCCBH31258		1	
Germany	CC374	ST268(3-2-2-2-3-1-5)	CP087340.1	DB008			
Tanzania	CC374	ST374(3-2-2-2-3-4-4)	CP053215.1	DT0544C			
			CP053216.1	DT0544C plasmid unnamed1			3
South Korea	CC374	ST155(3-2-2-2-44-4-4)	CP138324.1	JBAB			
India	CC374	ST374(3-2-2-2-3-4-4)	CP072305.1	KSK Sensitive			
unknown	CC374	ST729(3-3-2-2-3-1-4)	CP085788.1	RCH52		1	3
Singapore	CC374	ST374(3-2-2-2-3-4-4)	CP162300.1	SIMBA113			
Canada	CC374	ST155(3-2-2-2-44-4-4)	CP123854.1	WB4			
Japan	CC374	ST1548(3-2-2-2-3-1-4)	AP022238.1	WP8-W18-ESBL-11			
China	CC374	ST990(3-2-2-2-3-2-2)	CP134579.1	XH1034			
China	CC374	ST719(3-2-2-2-3-2-4)	CP134555.1	XH1044			
USA	CC78	ST78(25-3-6-2-28-1-29)	CP115637.1	2022CK-00211			5

Country	CC	ST(Pasteur)	Accession	strain	Frameshifted	Truncated	Complete
USA	CC78	ST78(25-3-6-2-28-1-29)	CP115639.1	2022CK-00241			5
USA	CC78	ST78(25-3-6-2-28-1-29)	CP115641.1	2022CK-00251			5
USA	CC78	ST78(25-3-6-2-28-1-29)	CP115643.1	2022CK-00317			5
USA	CC78	ST78(25-3-6-2-28-1-29)	CP115645.1	2022CK-00371			6
USA	CC78	ST78(25-3-6-2-28-1-29)	CP155721.1	2024CK-00246			5
USA	CC78	ST78(25-3-6-2-28-1-29)	CP155455.1	2024CK-00278			5
USA	CC78	ST78(25-3-6-2-28-1-29)	CP156933.1	2024CK-00546			5
USA	CC78	ST78(25-3-6-2-28-1-29)	CP167043.1	2024CK-00843		1	3
Belgium	CC78	ST78(25-3-6-2-28-1-29)	CP091373.1	AB21-VUB		1	4
Belgium	CC78	ST78(25-3-6-2-28-1-29)	CP091369.1	AB40-VUB		1	5
France	CC78	ST1077(25-3-6-2-28-1-2)	CP060029.1	AbCTX1	7	1	
France	CC78	ST78(25-3-6-2-28-1-29)	CP059729.1	AbCTX13	5	1	
France	CC78	N4(25-3-6-204#-28-1-29)	CP060505.1	AbCTX5	5	1	1
France	CC78	ST78(25-3-6-2-28-1-29)	CP060504.1	AbCTX9	6	2	3
USA	CC78	ST78(25-3-6-2-28-1-29)	CP039930.1	TG29392		1	3
USA	CC78	ST78(25-3-6-2-28-1-29)	CP039343.1	TG31302		1	3
USA	CC78	ST78(25-3-6-2-28-1-29)	CP039341.1	TG31986	1	1	2
China	CC149	ST46(5-12-11-2-14-9-14)	CP104295.1	DETAB-C9			
China	CC149	ST46(5-12-11-2-14-9-14)	CP077835.1	DETAB-P65			
Nepal	CC149	ST622(3-12-11-2-14-4-14)	AP014649.1	IOMTU433			
Nepal	CC149	N18(3-N-11-2-14-4-14)	AP031583.1	JUNP514			
			AP031584.1	JUNP514 plasmid Pjunp514			2
Nepal	CC149	ST149(3-12-11-2-14-9-14)	AP031585.1	JUNP543			
Malaysia	CC149	ST46(5-12-11-2-14-9-14)	CP043953.1	K09-14			
India	CC149	ST622(3-12-11-2-14-4-14)	CP072122.1	KSK1			
			CP072123.1	KSK1 plasmid p1KSK1		1	1
India	CC149	ST622(3-12-11-2-14-4-14)	CP072280.1	KSK10			
India	CC149	ST622(3-12-11-2-14-4-14)	CP072285.1	KSK11			
India	CC149	ST622(3-12-11-2-14-4-14)	CP072290.1	KSK18			
India	CC149	ST622(3-12-11-2-14-4-14)	CP072295.1	KSK19			
India	CC149	ST622(3-12-11-2-14-4-14)	CP072398.1	KSK2			
India	CC149	ST622(3-12-11-2-14-4-14)	CP072300.1	KSK20			
India	CC149	ST622(3-12-11-2-14-4-14)	CP072270.1	KSK6			
			CP072271.1	KSK6 plasmid p1KSK6		1	1
India	CC149	ST622(3-12-11-2-14-4-14)	CP072275.1	KSK7			
India	CC149	ST622(3-12-11-2-14-4-14)	CP040050.1	VB16141			
			CP040051.1	VB16141 plasmid unnamed1	1		1
China	CC149	ST46(5-12-11-2-14-9-14)	CP104297.1	ZHOU			
Nepal	CC15	ST15(6-6-8-2-3-5-4)	AP031579.1	JUNP406			
Ukraine	CC15	ST15(6-6-8-2-3-5-4)	CP179869.1	MRSN122172			
			CP179870.1	MRSN122172 plasmid p1MRSN122172		2	
USA	CC15	ST15(6-6-8-2-3-5-4)	CP179883.1	MRSN15091			
			CP179885.1	MRSN15091 plasmid p2MRSN15091			1
USA	CC15	ST15(6-6-8-2-3-5-4)	CP179872.1	MRSN15116			
			CP179873.1	MRSN15116 plasmid p1MRSN15116			2
Iraq	CC15	ST15(6-6-8-2-3-5-4)	CP179875.1	MRSN548102			
Iraq	CC15	ST15(6-6-8-2-3-5-4)	CP179880.1	MRSN548254			
			CP179882.1	MRSN548254 plasmid p2MRSN548254			1

Country	CC	ST(Pasteur)	Accession	strain	Frameshifted	Truncated	Complete
Chile	CC15	ST318(6-6-8-2-3-5-5)	CP076814.1	UC21460			
Chile	CC15	ST15(6-6-8-2-3-5-4)	CP076817.1	UC24137			
			CP076818.1	UC24137 plasmid p1UC24137			1
			CP076819.1	UC24137 plasmid p2UC24137			2
			CP179879.1	MRSN548102 plasmid p4MRSN548102			
USA	CC33	ST132(3-5-5-1-7-1-4)	CP174007.1	2024CK-01407			
China	CC33	ST138(3-3-7-26-7-1-4)	CP018332.1	A1296			
China	CC33	ST132(3-5-5-1-7-1-4)	CP083181.1	AB43			
China	CC33	ST138(3-3-7-26-7-1-4)	CP047973.1	DETAB-P2			
Germany	CC33	ST193(3-1-7-1-7-2-4)	CP087325.1	OC073			
Germany	CC33	ST33(3-5-7-1-7-1-4)	CP087328.1	OC074			
China	CC33	ST2034(3-5-7-1-7-2-4)	CP191655.1	W410			
Japan	CC33	ST213(3-3-7-1-7-1-4)	AP022077.1	WP4-W18-ESBL-11			
Colombia	CC85	ST464(5-2-4-1-3-4-4)	CP010397.1	6200			
Belgium	CC85	ST85(5-2-4-1-3-3-4)	CP091361.1	AB177-VUB			
Belgium	CC85	ST85(5-2-4-1-3-3-4)	CP091356.1	AB186-VUB			
Egypt	CC85	ST85(5-2-4-1-3-3-4)	CP160130.1	AB37-AUFP			
India	CC85	ST85(5-2-4-1-3-3-4)	CP038644.1	ACN21			1
			CP038646.1	ACN21 plasmid unnamed2	2	1	
Germany	CC85	ST85(5-2-4-1-3-3-4)	CP139836.1	B84			
Lebanon	CC85	ST85(5-2-4-1-3-3-4)	CP082952.1	CI300			
Germany	CC85	ST85(5-2-4-1-3-3-4)	CP087309.1	OC068			
Russia	CC203	ST203(3-4-2-2-7-1-2)	CP059386.1	36-1512			
USA	CC203	ST1543(3-4-2-2-7-1-4)	CP026125.1	ABNIH28			
Cambodia	CC203	ST203(3-4-2-2-7-1-2)	CP044356.1	CAM180-1			
Tanzania	CC203	ST239(1-4-2-2-7-1-4)	CP053218.1	DT01139C			
			CP053220.1	DT01139C plasmid unnamed2			1
Germany	CC203	ST58(13-4-2-2-7-1-2)	CP087300.1	OC061			
USA	CC203	ST2836(3-4-2-2-7-1-3)	CP169866.1	Rp779			
China	CC203	ST239(1-4-2-2-7-1-4)	CP134566.1	XH1038			
USA	CC406	N5(1-307-1-2-65-1-5)	CP043458.1	18WIARLN0024			3
USA	CC406	ST406(1-1-1-2-65-1-5)	CP046554.1	19WIARLN011_full			4
USA	CC406	ST406(1-1-1-2-65-1-5)	CP046552.1	19WIARLN021_full			3
USA	CC406	ST406(1-1-1-2-65-1-5)	CP046549.1	19WIARLN022_full			5
USA	CC406	ST406(1-1-1-2-65-1-5)	CP046546.1	19WIARLN023_full			3
USA	CC406	ST2512(1-2-1-2-65-1-5)	CP149838.1	2024CK-00130			4
USA	CC52	ST52(3-2-2-7-9-1-5)	CP015121.1	ab736			
USA	CC52	ST438(3-2-2-7-9-4-5)	CP065432.1	ATCC 17961			
USA	CC52	ST52(3-2-2-7-9-1-5)	CP045110.1	ATCC 19606			
Netherlands	CC52	ST52(3-2-2-7-9-1-5)	CP091334.1	ATCC19606-VUB			
China	CC52	ST52(3-2-2-7-9-1-5)	CP059474.1	M175-3			
			CP059476.1	M175-3 plasmid pM175-3.2			3
unknown	CC52	ST52(3-2-2-7-9-1-5)	CP064375.1	PartI-Abaumannii-RM8376			
unknown	CC103	ST773(7-114-2-1-7-1-4)	CP188291.1	Ab2210v1			
Ghana	CC103	ST103(7-3-2-1-7-1-4)	CP051875.1	Ab-B004d-c			2
Ghana	CC103	ST103(7-3-2-1-7-1-4)	CP051869.1	Ab-D10a-a			2
			CP051870.1	Ab-D10a-a plasmid pAb-D10a-a_1			2
Germany	CC103	ST103(7-3-2-1-7-1-4)	CP087312.1	OC059			

Country	CC	ST(Pasteur)	Accession	strain	Frameshifted	Truncated	Complete
Germany	CC103	ST103(7-3-2-1-7-1-4)	CP190363.1	Up398			
Egypt	CC158	ST158(41-42-13-1-5-4-14)	CP159763.1	AB11-AUFP		1	4
Belgium	CC158	ST158(41-42-13-1-5-4-14)	CP091372.1	AB32-VUB			
Egypt	CC158	ST158(41-42-13-1-5-4-14)	CP160032.1	AB44-AUFP		1	4
USA	CC158	ST342(41-60-13-1-5-4-14)	CP169869.1	Up280			
Czech Republic	CC109	ST345(26-4-2-2-9-2-5)	CP038262.1	EC			
Czech Republic	CC109	ST345(26-4-2-2-9-2-5)	CP038258.1	EH			
Chile	CC109	ST109(26-4-2-2-9-1-5)	CP076807.1	UC20804			3
			CP076808.1	UC20804 plasmid p1UC20804		1	2
			CP076809.1	UC20804 plasmid p2UC20804			1
USA	CC107	ST1142(34-4-37-1-5-6-36)	CP140420.1	2023CK-00893		1	2
Ghana	CC107	ST107(34-35-37-1-5-6-36)	CP051866.1	Ab-C63			
			CP051867.1	Ab-C63 plasmid pAb-C63_1			3
India	CC494	ST1422(3-3-2-5-29-1-4)	CP185772.1	Ab02			
unknown	CC494	ST494(3-3-2-5-4-1-4)	LT605059.1	NCTC7364			
Portugal	CC172	N9(1-16-2-6#-18-4-4)	CP178250.1	ACI		2	1
Argentina	CC172	ST172(1-16-2-6-18-4-4)	CP178253.1	A118			
China	CC639	N17(3-37-2-2-11-57-4)	CP150097.1	HAU425			
china	CC639	ST639(3-3-2-2-11-57-4)	CP006768.1	ZW85-1			
USA	-	ST499(5-2-39-2-3-1-5)	CP107577.1	1326359			1
USA	-	ST499(5-2-39-2-3-1-5)	CP107601.1	1326589		2	3
USA	-	ST499(5-2-39-2-3-1-5)	CP107603.1	1326595		2	3
USA	-	ST499(5-2-39-2-3-1-5)	CP107579.1	1326525-1		1	3
USA	-	ST499(5-2-39-2-3-1-5)	CP107581.1	1326525-2		1	3
USA	-	ST499(5-2-39-2-3-1-5)	CP107583.1	1326525-3		1	3
USA	-	ST499(5-2-39-2-3-1-5)	CP107585.1	1326527-1		2	3
USA	-	ST499(5-2-39-2-3-1-5)	CP107595.1	1326581-1			1
USA	-	ST499(5-2-39-2-3-1-5)	CP107597.1	1326581-2			2
USA	-	ST499(5-2-39-2-3-1-5)	CP107608.1	1326924-2		2	3
USA	-	ST499(5-2-39-2-3-1-5)	CP107610.1	1326924-3		2	3
USA	-	ST499(5-2-39-2-3-1-5)	CP107612.1	1326927-1		2	3
USA	-	ST499(5-2-39-2-3-1-5)	CP107614.1	1326927-2		2	3
USA	-	ST499(5-2-39-2-3-1-5)	CP115632.1	2022CK-00063		2	2
USA	-	ST499(5-2-39-2-3-1-5)	CP115623.1	2022CK-00340			3
USA	-	ST499(5-2-39-2-3-1-5)	CP115621.1	2022CK-00480			3
USA	-	ST499(5-2-39-2-3-1-5)	CP166843.1	2024CK-00841			3
USA	-	ST499(5-2-39-2-3-1-5)	CP166841.1	2024CK-00842			5
USA	-	ST499(5-2-39-2-3-1-5)	CP169592.1	2024CK-01227			1
USA	-	ST499(5-2-39-2-3-1-5)	CP174438.1	2024CK-01444		1	1
USA	-	ST499(5-2-39-2-3-1-5)	CP174436.1	2024CK-01445			2
USA	-	ST499(5-2-39-2-3-1-5)	CP180124.1	2024CK-01502			1
USA	-	ST499(5-2-39-2-3-1-5)	CP180122.1	2024CK-01509			1
USA	-	ST499(5-2-39-2-3-1-5)	CP180040.1	2024CK-01684			4
USA	-	ST499(5-2-39-2-3-1-5)	CP081137.1	ARLG_6420		1	2
unknown	-	ST499(5-2-39-2-3-1-5)	CP169773.1	Hv431			
USA	-	ST499(5-2-39-2-3-1-5)	CP169779.1	Hv635			
USA	-	ST499(5-2-39-2-3-1-5)	CP169782.1	Hv640			
USA	-	ST499(5-2-39-2-3-1-5)	CP169795.1	Hv780			

Country	CC	ST(Pasteur)	Accession	strain	Frameshifted	Truncated	Complete
USA	-	ST499(5-2-39-2-3-1-5)	CP169798.1	Hv781			
USA	-	ST499(5-2-39-2-3-1-5)	CP169801.1	Hv782			
USA	-	ST499(5-2-39-2-3-1-5)	CP190370.1	Up425			
USA	-	ST499(5-2-39-2-3-1-5)	CP033876.1	WU_164			
China	-	ST164(40-3-7-2-40-4-4)	CP191668.1	AB1801			
China	-	ST164(40-3-7-2-40-4-4)	CP188287.1	Ab2211v1			
China	-	ST164(40-3-7-2-40-4-4)	CP188281.1	Ab2258v1			
China	-	ST164(40-3-7-2-40-4-4)	CP188269.1	Ab2406v1			
China	-	ST164(40-3-7-2-40-4-4)	CP161817.1	DETAB-P462			
China	-	ST164(40-3-7-2-40-4-4)	CP088895.1	DETAB-R21			
Nepal	-	ST164(40-3-7-2-40-4-4)	AP031581.1	JUNP496			1
China	-	ST164(40-3-7-2-40-4-4)	CP178406.1	L4773hy			
China	-	ST164(40-3-7-2-40-4-4)	CP178412.1	L4793hy			
China	-	ST164(40-3-7-2-40-4-4)	CP178418.1	L4796hy			
France	-	ST164(40-3-7-2-40-4-4)	CP169811.1	Lv371			
			CP169812.1	Lv371 plasmid unnamed1			3
China	-	ST164(40-3-7-2-40-4-4)	CP144242.1	SRM3			
China	-	ST164(40-3-7-2-40-4-4)	CP191658.1	W398			
China	-	ST164(40-3-7-2-40-4-4)	CP088894.1	XH1935			
Belgium	-	ST636(2-1-2-2-2-1-1)	CP091376.1	AB14-VUB		1	7
Belgium	-	ST636(2-1-2-2-2-1-1)	CP091362.1	AB176-VUB		1	6
Belgium	-	ST636(2-1-2-2-2-1-1)	CP091355.1	AB187-VUB		1	10
Belgium	-	ST636(2-1-2-2-2-1-1)	CP091354.1	AB188-VUB		1	7
Belgium	-	ST636(2-1-2-2-2-1-1)	CP091374.1	AB20-VUB		1	8
Belgium	-	ST636(2-1-2-2-2-1-1)	CP091377.1	AB9-VUB			4
Germany	-	ST636(2-1-2-2-2-1-1)	CP087354.1	DB002		1	6
Germany	-	ST636(2-1-2-2-2-1-1)	CP087351.1	DB003		1	6
Germany	-	ST636(2-1-2-2-2-1-1)	CP087344.1	DB007		1	6
France	-	ST636(2-1-2-2-2-1-1)	CP169791.1	Hv770		1	5
Netherlands	-	ST32(1-1-2-2-3-4-4)	CP133717.1	Ab1			
Unknown	-	ST32(1-1-2-2-3-4-4)	CP027183.1	AR_0052			
			CP027184.1	AR_0052 plasmid unnamed2	1		3
Unknown	-	ST32(1-1-2-2-3-4-4)	CP027178.1	AR_0070			
			CP027179.1	AR_0070 plasmid unnamed1			3
Netherlands	-	ST32(1-1-2-2-3-4-4)	CP133712.1	CRAb1			
			CP133713.1	CRAb1 plasmid unnamed01			2
Netherlands	-	ST32(1-1-2-2-3-4-4)	CP133706.1	CRAb2			
			CP133707.1	CRAb2 plasmid unnamed01			2
Germany	-	ST32(1-1-2-2-3-4-4)	CP087335.1	DB053			
France	-	ST32(1-1-2-2-3-4-4)	CP169859.1	Rp772			
China	-	ST32(1-1-2-2-3-4-4)	CP191662.1	W20			
			CP191663.1	W20 plasmid W20_pNDM			1
China	-	ST40(1-2-2-2-5-1-14)	CP091173.1	AB5116			
China	-	ST40(1-2-2-2-5-1-14)	CP102762.1	AOR07-BL			
			CP102764.1	AOR07-BL plasmid pAOR07BL-2			2
USA	-	ST40(1-2-2-2-5-1-14)	CP113442.1	C9			
China	-	ST40(1-2-2-2-5-1-14)	CP087594.1	SHOU-Ab01			
China	-	ST40(1-2-2-2-5-1-14)	CP134601.1	XH1022			

Country	CC	ST(Pasteur)	Accession	strain	Frameshifted	Truncated	Complete
China	-	ST40(1-2-2-2-5-1-14)	CP134586.1	XH1026			
India	-	ST126(3-2-7-2-7-1-3)	CP098791.1	280820			
			CP098792.1	280820 plasmid p1VB280820			3
Malaysia	-	ST126(3-2-7-2-7-1-3)	CP059300.1	AC1633			
USA	-	ST126(3-2-7-2-7-1-3)	LN865143.1	CIP70.10			
France	-	ST126(3-2-7-2-7-1-3)	LN997846.1	R2091			
India	-	ST126(3-2-7-2-7-1-3)	CP098795.1	VB280821			
			CP098796.1	VB280821 plasmid p1VB280821			3
China	-	ST150(39-2-2-2-4-27-4)	CP133052.1	A1014			
China	-	ST150(39-2-2-2-4-27-4)	CP169375.1	H3			
UNKNOW	-	ST150(39-2-2-2-4-27-4)	CP144465.1	PUMA0246			
Singapore	-	ST150(39-2-2-2-4-27-4)	CP161999.1	SIMBA061			
China	-	ST150(39-2-2-2-4-27-4)	CP046536.1	XL380			
USA	-	ST16(7-7-2-2-8-4-4)	CP107593.1	1326580			
USA	-	ST16(7-7-2-2-8-4-4)	CP107599.1	1326584			
USA	-	ST16(7-7-2-2-8-4-4)	CP107616.1	1326932			
China	-	ST16(7-7-2-2-8-4-4)	CP065051.1	2016GDAB1			
			CP065052.1	2016GDAB1 plasmid p5637			2
China	-	ST16(7-7-2-2-8-4-4)	CP044519.1	29FS20			
			CP044520.1	29FS20 plasmid p29FS20-1			3
Belgium	-	ST215(27-2-7-2-2-1-2)	CP091338.1	AB231-VUB		1	4
China	-	ST215(27-2-7-2-2-1-2)	CP134589.1	XH1024		2	9
China	-	ST215(27-2-7-2-2-1-2)	CP014540.1	XH857		2	9
China	-	ST331(3-2-2-2-7-2-5)	CP121365.1	Ab_8_4			
China	-	ST331(3-2-2-2-7-2-5)	CP121370.1	LRB			
China	-	ST331(3-2-2-2-7-2-5)	CP121375.1	LRT			
Turkey	-	N10(1-2-2-1-11-2-4)	CP171396.1	T14			
Turkey	-	N10(1-2-2-1-11-2-4)	CP171398.1	T24			
China	-	ST108(35-1-11-7-9-25-2)	CP046898.1	A1429			
China	-	ST108(35-1-11-7-9-25-2)	CP134583.1	XH1032			
China	-	ST1093(1-158-2-2-165-1-2)	CP044517.1	31FS3-2			3
China	-	ST1093(1-158-2-2-165-1-2)	CP054560.1	YC103			
India	-	ST1547(13-3-7-3-7-1-16)	CP021347.1	B8300			
Australia	-	ST1547(13-3-7-3-7-1-16)	CP042556.1	E47			
			CP042557.1	E47 plasmid pE47_001			3
Chile	-	ST162(3-2-2-2-2-4-8)	CP076804.1	UC24371			
			CP076805.1	UC24371 plasmid p1UC24371			1
Chile	-	ST162(3-2-2-2-2-4-8)	CP076801.1	UC25604			
			CP076802.1	UC25604 plasmid p1UC25604			1
Unknown	-	ST229(3-3-51-2-28-1-3)	CP026761.1	AR_0078			9
USA	-	ST229(3-3-51-2-28-1-3)	CP169832.1	Lv648			9
Australia	-	ST267(12-37-2-2-3-2-14)	CP003967.2	D1279779			
France	-	ST267(12-37-2-2-3-2-14)	LN868200.1	R2090			
China	-	ST338(8-5-5-26-13-1-2)	CP191669.1	AB0614			
China	-	ST338(8-5-5-26-13-1-2)	CP191648.1	W76			
USA	-	ST647(3-3-6-2-51-1-29)	CP059547.1	10_3			
USA	-	ST647(3-3-6-2-51-1-29)	CP059546.1	10_4			
Ghana	-	ST1472(3-1-5-26-13-2-3)	CP051862.1	Ab-C102			

Country	CC	ST(Pasteur)	Accession	strain	Frameshifted	Truncated	Complete
			CP051863.1	Ab-C102 plasmid pAb-C102_1			2
China	-	ST2253(3-4-2-2-5-2-4)	CP084297.1	LHC22-2			
			CP084298.1	LHC22-2 plasmid pLHC22-2-tetX-162k			1
Australia	-	ST111(3-3-2-2-4-8-12)	CP110462.1	RBH2			1
			CP110466.1	RBH2 plasmid pRBH2-4			1
USA	-	ST274(40-2-2-2-9-1-36)	CP096812.1	51248			
unknown	-	N16(3-3-6-2-102-4-4)	OZ197095.1	23S01404-6			
Poland	-	ST309(12-1-2-2-9-1-5)	CP113069.1	29D2			
Poland	-	ST690(3-3-2-1-7-2-14)	CP113077.1	86II/2C			
China	-	ST152(8-1-5-3-6-2-3)	CP132915.1	A207			
Canada	-	ST638(100-3-14-1-7-1-4)	CP009256.1	AB031			
India	-	ST479(3-3-11-2-44-4-8)	CP185767.1	AB07			
India	-	ST2276(3-2-2-34-5-1-14)	CP185766.1	AB08			
China	-	ST2266(8-1-56-3-7-1-4)	CP103338.1	AB105			
China	-	ST282(3-3-2-6-52-2-14)	CP123993.1	Ab4294			
China	-	ST1336(1-3-40-2-7-1-1)	CP149800.1	AB7276			
France	-	ST2532(3-1-5-3-3-1-2)	CP136183.1	ABO21-A001			
France	-	ST1384(1-3-194-2-4-4-5)	CP136178.1	ABO21-A045			
France	-	ST578(97-3-13-1-4-4-14)	CP136173.1	ABO21-A049			
France	-	ST2247(25-3-7-2-5-4-5)	CP136172.1	ABO21-A051			
India	-	ST1545(3-1-7-5-3-1-4)	CP021342.1	B8342			
Australia	-	ST1546(3-3-15-5-71-1-36)	CP012587.1	CA-17			
Malaysia	-	ST142(13-4-40-1-42-1-16)	CP060994.1	CAb-65			
China	-	ST1554(3-3-2-79-3-4-4)	CP072526.1	DETAB-E227			
China	-	ST221(3-1-2-1-18-1-48)	CP073060.1	DETAB-P39			
India	-	N11(13-3#-7-26-7-1-29)	CP027704.2	DS002			
USA	-	ST738(3-3-105-6-4-2-5)	CP091333.1	DSM30011-VUB			
Finland	-	ST648(1-3-11-5-111-1-14)	CP059542.1	E-011922			
Finland	-	ST649(27-3-2-2-5-58-5)	CP061705.1	E-072658			
China	-	ST477(26-2-6-2-5-1-5)	CP142019.1	EMB-1			
USA	-	ST57(1-3-17-5-3-1-14)	CP033768.1	FDAARGOS_533			
USA	-	ST1542(1-4-5-3-6-2-3)	CP033754.1	FDAARGOS_540			
Australia	-	ST49(3-3-6-2-3-1-5)	CP041587.1	J9			
Saudi Arabia	-	ST1572(2-2-2-2-5-2-1)	CP121612.1	JAB117			5
Germany	-	ST54(12-3-18-2-17-4-5)	CP087331.1	LMG994			
China	-	ST866(3-2-2-30-3-74-3)	CP058729.1	M164-3			
USA	-	ST1197(1-4-2-2-7-58-2)	CP028138.1	NCIMB 8209			
Germany	-	ST400(3-3-55-2-66-1-5)	CP087298.1	OC070			
Japan	-	N7(13-4-5-3-6-1-29)	AP024802.1	OCU Ac18			
Japan	-	ST412(1-52-2-2-67-4-5)	AP023077.1	OCU_Ac16a			
unknown	-	ST294(40-3-2-2-4-35-4)	CP043180.1	PG20180064			
UNKNOW	-	N12(3-1#-7-1-9-1-4)	CP151078.1	PUMA0052			
Canada	-	ST2252(5-4-2-1-3-1-5)	CP062919.1	Res13-Abat-PEA21-P4-01-A			
Australia	-	ST350(3-3-2-2-5-2-5)	CP127906.1	SAAb472			
USA	-	N6(50-3#-6#-1-3-4-4)	CP064292.1	SD	1		1
South Korea	-	ST756(12-1-7-1-7-2-29)	CP137055.1	SNUBHAB0137			
India	-	ST285(1-52-2-2-9-4-2)	CP040080.1	SP304			
China	-	N19(5-1-101-2-5-1-5)	CP163382.1	Y03			
