# Supplementary material for: Impact of IS26 mobilization on genetic manipulation of multidrug-resistant Acinetobacter baumannii
Source: Front Microbiol. 2025 Oct 8;16:1689239. doi: 10.3389/fmicb.2025.1689239 (PMC12540391; doi:10.3389/fmicb.2025.1689239)
Supplement: Supplementary file 3 [file Data_Sheet_3.pdf]

Table S3. Distributions of intact IS*26* in NCBI *A.baumannii* genomes

**Clonal Cluster**	**MLST(Pasteur)***	**IS*26* in Chromosome/ Plasmid**				**SUM**	**IS26 (+)**	**Copy NO. (range)**	**CC SUM**	**CC IS26(+)**
		+/+	+/-	-/+	-/-					
IC2/CC2	ST2(2-2-2-2-2-2-2)	10	464			**474**	474	1~21	510	509
	ST724(2-2-2-2-2-2-5)		7			**7**	7	9		
	ST570(2-2-2-2-2-2-4)		5			**5**	5	7，8		
	N8(2-2-2-2-2-2$^{\#}$-2)		3			**3**	3	5~7		
	ST1579(2-229-2-2-2-2-2)		3			**3**	3	2		
	ST195(2-2-2-2-2-1-2)		3			**3**	3	3~6		
	ST187(2-2-2-2-2-2-43)		2			**2**	2	1，4		
	N1(2-307-2-2-2-2-2)$^{\$}$			1		**1**	1	1		
	N2(2-307$^{\#}$-2-2-2-2-460)	1				**1**	1	3		
	ST104(2-2-2-2-2-2-14)		1			**1**	1	5		
	ST1544(2-2-2-2-2-129-2)		1			**1**	1	8		
	ST1550(2-226-2-2-2-2-2)		1			**1**	1	3		
	ST1555(2-2-181-2-2-2-2-2)		1			**1**	1	5		
	ST1580(2-230-2-2-2-2-2)		1			**1**	1	5		
	ST2716(2-2-2-2-615-2-2)		1			**1**	1	2		
	ST45(2-6-2-2-2-2-2)		1			**1**	1	8		
	ST571(2-2-2-2-104-2-4)$^{\$}$				1	**1**	0			
	ST604(2-2-2-55-2-2-2)		1			**1**	1	6		
	ST922(2-2-2-75-2-2-2)		1			**1**	1	5		
	ST98(1-2-2-2-2-2-2)		1			**1**	1	5		
IC1/CC1	ST1(1-1-1-1-5-1-1)	6	30		18	**54**	36	1~153	56	38
	ST20(3-1-1-1-5-1-1)		1			**1**	1	3		
	ST81(1-1-1-1-5-1-2)	1				**1**	1	4		
IC7/CC25	ST25(3-3-2-4-7-2-4)		2	7	26	**35**	9	1~4	38	11
	N14(3-3-2-4$^{\#}$-7-2-4)			1		**1**	1	1		
	N15(3-3-2-4-7-2-N)			1		**1**	1	2		
	ST307(3-3-2-4-7-2-30)				1	**1**	0			
IC8/CC23	ST10(1-3-2-1-4-4-4)		2	6	12	**20**	8	1~3	28	11
	ST23(1-3-10-1-4-4-4)				4	**4**	0			
	ST575(1-3-2-1-4-4-92)			1	1	**2**	1	1		
	ST1512(1-3-10-1-4-1-4)			1		**1**	1	1		
	ST256(1-3-10-1-2-4-4)		1			**1**	1	2		
IC5/CC79	ST79(26-2-2-2-29-4-5)	1	10	1		**12**	12	1~5	24	22
	ST422(26-72-2-2-29-4-5)		8		2	**10**	8	3		
	ST156(26-2-2-2-29-4-4)		1			**1**	1	1		
	ST298(1-2-2-2-29-4-5)		1			**1**	1	2		
CC374	ST437(3-2-2-2-30-4-28)				7	**7**	0		21	2
	ST374(3-2-2-2-3-4-4)			1	2	**3**	1	3		
	ST155(3-2-2-2-44-4-4)				2	**2**	0			
	N13(3-2-2-2-44-1-4)				1	**1**	0			
	N3(3$^{\#}$-2-2-2-3-4-4)				1	**1**	0			
	ST1548(3-2-2-2-3-1-4)				1	**1**	0			
	ST268(3-2-2-2-3-1-5)				1	**1**	0			
	ST719(3-2-2-2-3-2-4)				1	**1**	0			

Clonal Cluster	MLST(Pasteur)*	IS*26* in Chromosome/ Plasmid				SUM	IS26 (+)	Copy NO. (range)	CC SUM	CC IS26(+)
		+/+	+/-	-/+	-/-					
	ST729(3-3-2-2-3-1-4)		1			**1**	1	3		
	ST77(3-2-2-2-3-4-28)				1	**1**	0			
	ST825(3-2-2-2-7-4-4)				1	**1**	0			
	ST990(3-2-2-2-3-2-2)				1	**1**	0			
IC6/CC78	ST78(25-3-6-2-28-1-29)		15		1[$]	**16**	15	1~6	18	16
	N4(25-3-6-204[#]-28-1-29)		1			**1**	1	1		
	ST1077(25-3-6-2-28-1-2)				1[$]	**1**	0			
CC149	ST622(3-12-11-2-14-4-14)			3	8	**11**	3	1	17	4
	ST46(5-12-11-2-14-9-14)				4	**4**	0			
	N18(3-N-11-2-14-4-14)			1		**1**	1	2		
	ST149(3-12-11-2-14-9-14)				1	**1**	0			
IC4/CC15	ST15(6-6-8-2-3-5-4)			6	1	**7**	6	1~3	8	6
	ST318(6-6-8-2-3-5-5)				1	**1**	0			
CC33	ST132(3-5-5-1-7-1-4)				2	**2**	0		8	0
	ST138(3-3-7-26-7-1-4)				2	**2**	0			
	ST193(3-1-7-1-7-2-4)				1	**1**	0			
	ST2034(3-5-7-1-7-2-4)				1	**1**	0			
	ST213(3-3-7-1-7-1-4)				1	**1**	0			
	ST33(3-5-7-1-7-1-4)				1	**1**	0			
IC9/CC85	ST85(5-2-4-1-3-3-4)		1		6	**7**	1	1	8	1
	ST464(5-2-4-1-3-4-4)				1	**1**	0			
CC203	ST203(3-4-2-2-7-1-2)				2	**2**	0		7	1
	ST239(1-4-2-2-7-1-4)			1	1	**2**	1	1		
	ST1543(3-4-2-2-7-1-4)				1	**1**	0			
	ST2836(3-4-2-2-7-1-3)				1	**1**	0			
	ST58(13-4-2-2-7-1-2)				1	**1**	0			
CC406	ST406(1-1-1-2-65-1-5)		4			**4**	4	3~5	6	6
	N5(1-307-1-2-65-1-5)		1			**1**	1	3		
	ST2512(1-2-1-2-65-1-5)		1			**1**	1	4		
CC52	ST52(3-2-2-7-9-1-5)			1	4	**5**	1	3	6	1
	ST438(3-2-2-7-9-4-5)				1	**1**	0			
CC103	ST103(7-3-2-1-7-1-4)	1	1		2	**4**	2	2~4	5	2
	ST773(7-114-2-1-7-1-4)				1	**1**	0			
CC158	ST158(41-42-13-1-5-4-14)			2	1	**3**	2	4	4	2
	ST342(41-60-13-1-5-4-14)				1	**1**	0			
CC109	ST345(26-4-2-2-9-2-5)				2	**2**	0		3	1
	ST109(26-4-2-2-9-1-5)	1				**1**	1	8		
CC107	ST107(34-35-37-1-5-6-36)			1		**1**	1	3	2	2
	ST1142(34-4-37-1-5-6-36)		1			**1**	1	2		
CC494	ST1422(3-3-2-5-29-1-4)				1	**1**	0		2	0
	ST494(3-3-2-5-4-1-4)				1	**1**	0			
CC172	ST172(1-16-2-6-18-4-4)				1	**1**	0		2	1
	N9(1-16-2-6[#]-18-4-4)		1			**1**	1	1		
CC639	ST639(3-3-2-2-11-57-4)				1	**1**	0		2	0
	N17(3-37-2-2-11-57-4)				1	**1**	0			
-	ST499(5-2-39-2-3-1-5)		25		8	**33**	25	1~5		

Clonal Cluster	MLST(Pasteur)*	IS*26* in Chromosome/ Plasmid				SUM	IS26 (+)	Copy NO. (range)	CC SUM	CC IS26(+)
		+/+	+/-	-/+	-/-					
-	ST164(40-3-7-2-40-4-4)		1	1	12	**14**	2	1, 3		
-	ST636(2-1-2-2-2-1-1)		10			**10**	10	4~10		
-	ST32(1-1-2-2-3-4-4)			5	3	**8**	5	1~3		
-	ST40(1-2-2-2-5-1-14)			1	5	**6**	1			
-	ST126(3-2-7-2-7-1-3)			2	3	**5**	2	3		
-	ST150(39-2-2-2-4-27-4)				5	**5**	0			
-	ST16(7-7-2-2-8-4-4)			2	3	**5**	2	2, 3		
-	ST215(27-2-7-2-2-1-2)		3			**3**	3	4~9		
-	ST331(3-2-2-2-7-2-5)				3	**3**	0			
-	N10(1-2-2-1-11-2-4)				2	**2**	0			
-	ST108(35-1-11-7-9-25-2)				2	**2**	0			
-	ST1093(1-158-2-2-165-1-2)		1		1	**2**	1	3		
-	ST1547(13-3-7-3-7-1-16)			1	1	**2**	1	3		
-	ST162(3-2-2-2-2-4-8)			2		**2**	2	1		
IC3/-	ST229(3-3-51-2-28-1-3)		2			**2**	2	9		
-	ST267(12-37-2-2-3-2-14)				2	**2**	0			
-	ST338(8-5-5-26-13-1-2)				2	**2**	0			
-	ST647(3-3-6-2-51-1-29)				2	**2**	0			
-	N11(13-3#-7-26-7-1-29)				1	**1**	0			
-	N12(3-1#-7-1-9-1-4)				1	**1**	0			
-	N16(3-3-6-2-102-4-4)				1	**1**	0			
-	N19(5-1-101-2-5-1-5)				1	**1**	0			
-	N6(50-3#-6#-1-3-4-4)			1		**1**	1	1		
-	N7(13-4-5-3-6-1-29)				1	**1**	0			
-	ST111(3-3-2-2-4-8-12)	1				**1**	1	2		
-	ST1197(1-4-2-2-7-58-2)				1	**1**	0			
-	ST1336(1-3-40-2-7-1-1)				1	**1**	0			
-	ST1384(1-3-194-2-4-4-5)				1	**1**	0			
-	ST142(13-4-40-1-42-1-16)				1	**1**	0			
-	ST1472(3-1-5-26-13-2-3)			1		**1**	1	2		
-	ST152(8-1-5-3-6-2-3)				1	**1**	0			
-	ST1542(1-4-5-3-6-2-3)				1	**1**	0			
-	ST1545(3-1-7-5-3-1-4)				1	**1**	0			
-	ST1546(3-3-15-5-71-1-36)				1	**1**	0			
-	ST1554(3-3-2-79-3-4-4)				1	**1**	0			
-	ST1572(2-2-2-2-5-2-1)		1			**1**	1	5		
-	ST221(3-1-2-1-18-1-48)				1	**1**	0			
-	ST2247(25-3-7-2-5-4-5)				1	**1**	0			
-	ST2252(5-4-2-1-3-1-5)				1	**1**	0			
-	ST2253(3-4-2-2-5-2-4)			1		**1**	1	1		
-	ST2266(8-1-56-3-7-1-4)				1	**1**	0			
-	ST2276(3-2-2-34-5-1-14)				1	**1**	0			
-	ST2532(3-1-5-3-3-1-2)				1	**1**	0			
-	ST274(40-2-2-2-9-1-36)				1	**1**	0			
-	ST282(3-3-2-6-52-2-14)				1	**1**	0			
-	ST285(1-52-2-2-9-4-2)				1	**1**	0			

Clonal Cluster	MLST(Pasteur)*	IS*26* in Chromosome/ Plasmid				SUM	IS26 (+)	Copy NO. (range)	CC SUM	CC IS26(+)
		+/+	+/-	-/+	-/-					
-	ST294(40-3-2-2-4-35-4)				1	**1**	0			
-	ST309(12-1-2-2-9-1-5)				1	**1**	0			
-	ST350(3-3-2-2-5-2-5)				1	**1**	0			
-	ST400(3-3-55-2-66-1-5)				1	**1**	0			
-	ST412(1-52-2-2-67-4-5)				1	**1**	0			
-	ST477(26-2-6-2-5-1-5)				1	**1**	0			
-	ST479(3-3-11-2-44-4-8)				1	**1**	0			
-	ST49(3-3-6-2-3-1-5)				1	**1**	0			
-	ST54(12-3-18-2-17-4-5)				1	**1**	0			
-	ST57(1-3-17-5-3-1-14)				1	**1**	0			
-	ST578(97-3-13-1-4-4-14)				1	**1**	0			
-	ST638(100-3-14-1-7-1-4)				1	**1**	0			
-	ST648(1-3-11-5-111-1-14)				1	**1**	0			
-	ST649(27-3-2-2-5-58-5)				1	**1**	0			
-	ST690(3-3-2-1-7-2-14)				1	**1**	0			
-	ST738(3-3-105-6-4-2-5)				1	**1**	0			
-	ST756(12-1-7-1-7-2-29)				1	**1**	0			
-	ST866(3-2-2-30-3-74-3)				1	**1**	0			
	SUM	22	624	52	233	931	698			

* New STs were designated using Arabic numerals, initiated by the letter N. For the ones no distinct alleles were assigned at the seven loci, the closest alleles were selected and marked by a “#”.

$ All copies of IS*26* within the chromosome exhibit frameshift mutations.
