# Supplementary material for: Impact of IS26 mobilization on genetic manipulation of multidrug-resistant Acinetobacter baumannii
Source: Front Microbiol. 2025 Oct 8;16:1689239. doi: 10.3389/fmicb.2025.1689239 (PMC12540391; doi:10.3389/fmicb.2025.1689239)
Supplement: Supplementary file 4 [file Data_Sheet_4.pdf]

Table S4. Distributions of IS*Aba1* in NCBI *A.baumannii* genomes

Clonal Cluster	MLST(Pasteur)*	IS*Aba1* in Chromosome/ Plasmid				SUM	ISAba1(+)	CC SUM	CC ISAba1(+)
		+/+	+/-	-/+	-/-				
IC2/CC2	ST2(2-2-2-2-2-2-2)	99	371	1	3	**474**	471	510	505
	ST724(2-2-2-2-2-2-5)	7				**7**	7		
	ST570(2-2-2-2-2-2-4)	1	4			**5**	5		
	N8(2-2-2-2-2-2#-2)		3			**3**	3		
	ST1579(2-229-2-2-2-2-2)		3			**3**	3		
	ST195(2-2-2-2-2-1-2)	1	2			**3**	3		
	ST187(2-2-2-2-2-2-43)		2			**2**	2		
	N1(2-307-2-2-2-2-2)	1				**1**	1		
	N2(2-307#-2-2-2-2-460)	1				**1**	1		
	ST104(2-2-2-2-2-2-14)	1				**1**	1		
	ST1544(2-2-2-2-2-129-2)	1				**1**	1		
	ST1550(2-226-2-2-2-2-2)				1	**1**	0		
	ST1555(2-2-181-2-2-2-2-2)		1			**1**	1		
	ST1580(2-230-2-2-2-2-2)		1			**1**	1		
	ST2716(2-2-2-2-615-2-2)		1			**1**	1		
	ST45(2-6-2-2-2-2-2)				1	**1**	0		
	ST571(2-2-2-2-104-2-4)		1			**1**	1		
	ST604(2-2-2-55-2-2-2)		1			**1**	1		
	ST922(2-2-2-75-2-2-2)		1			**1**	1		
	ST98(1-2-2-2-2-2-2)		1			**1**	1		
IC1/CC1	ST1(1-1-1-1-5-1-1)	7	39		8	**54**	46	56	48
	ST20(3-1-1-1-5-1-1)		1			**1**	1		
	ST81(1-1-1-1-5-1-2)	1				**1**	1		
IC7/CC25	ST25(3-3-2-4-7-2-4)	30	3	2		**35**	35	38	38
	N14(3-3-2-4#-7-2-4)	1				**1**	1		
	N15(3-3-2-4-7-2-N)	1				**1**	1		
	ST307(3-3-2-4-7-2-30)	1				**1**	1		
IC8/CC23	ST10(1-3-2-1-4-4-4)	6	8	4	2	**20**	18	28	26
	ST23(1-3-10-1-4-4-4)	1	3			**4**	4		
	ST575(1-3-2-1-4-4-92)	1	1			**2**	2		
	ST1512(1-3-10-1-4-1-4)	1				**1**	1		
	ST256(1-3-10-1-2-4-4)		1			**1**	1		
IC5/CC79	ST79(26-2-2-2-29-4-5)	3	8		1	**12**	11	24	23
	ST422(26-72-2-2-29-4-5)		10			**10**	10		
	ST156(26-2-2-2-29-4-4)		1			**1**	1		
	ST298(1-2-2-2-29-4-5)		1			**1**	1		
CC374	ST437(3-2-2-2-30-4-28)		4	1	2	**7**	5	21	9
	ST374(3-2-2-2-3-4-4)		1	1	1	**3**	2		
	ST155(3-2-2-2-44-4-4)				2	**2**	0		
	N13(3-2-2-2-44-1-4)				1	**1**	0		
	N3(3#-2-2-2-3-4-4)				1	**1**	0		
	ST1548(3-2-2-2-3-1-4)				1	**1**	0		
	ST268(3-2-2-2-3-1-5)				1	**1**	0		
	ST719(3-2-2-2-3-2-4)			1		**1**	1		
	ST729(3-3-2-2-3-1-4)		1			**1**	1		
	ST77(3-2-2-2-3-4-28)				1	**1**	0		
	ST825(3-2-2-2-7-4-4)				1	**1**	0		
	ST990(3-2-2-2-3-2-2)				1	**1**	0		
IC6/CC78	ST78(25-3-6-2-28-1-29)	2	14			**16**	16	18	18
	N4(25-3-6-204#-28-1-29)		1			**1**	1		
	ST1077(25-3-6-2-28-1-2)		1			**1**	1		
CC149	ST622(3-12-11-2-14-4-14)	4	6		1	**11**	10	17	15
	ST46(5-12-11-2-14-9-14)	3			1	**4**	3		
	N18(3-N-11-2-14-4-14)		1			**1**	1		
	ST149(3-12-11-2-14-9-14)		1			**1**	1		
IC4/CC15	ST15(6-6-8-2-3-5-4)	3	4			**7**	7	8	8
	ST318(6-6-8-2-3-5-5)		1			**1**	1		
CC33	ST132(3-5-5-1-7-1-4)				2	**2**	0	8	1
	ST138(3-3-7-26-7-1-4)			1	1	**2**	1		
	ST193(3-1-7-1-7-2-4)				1	**1**	0		
	ST2034(3-5-7-1-7-2-4)				1	**1**	0		
	ST213(3-3-7-1-7-1-4)				1	**1**	0		
	ST33(3-5-7-1-7-1-4)				1	**1**	0		

Clonal Cluster	MLST(Pasteur)*	IS*Aba1* in Chromosome/ Plasmid				SUM	ISAba1(+)	CC SUM	CC ISAba1(+)
		+/+	+/-	-/+	-/-				
IC9/CC85	ST85(5-2-4-1-3-3-4)	4	2	1		**7**	7	8	7
	ST464(5-2-4-1-3-4-4)				1	**1**	0		
CC203	ST203(3-4-2-2-7-1-2)			1	1	**2**	1	7	3
	ST239(1-4-2-2-7-1-4)			1	1	**2**	1		
	ST1543(3-4-2-2-7-1-4)				1	**1**	0		
	ST2836(3-4-2-2-7-1-3)				1	**1**	0		
	ST58(13-4-2-2-7-1-2)	1				**1**	1		
CC406	ST406(1-1-1-2-65-1-5)		4			**4**	4	6	6
	N5(1-307-1-2-65-1-5)	1				**1**	1		
	ST2512(1-2-1-2-65-1-5)		1			**1**	1		
CC52	ST52(3-2-2-7-9-1-5)			1	4	**5**	1	6	1
	ST438(3-2-2-7-9-4-5)				1	**1**	0		
CC103	ST103(7-3-2-1-7-1-4)	1			3	**4**	1	5	1
	ST773(7-114-2-1-7-1-4)				1	**1**	0		
CC158	ST158(41-42-13-1-5-4-14)	2		1		**3**	3	4	3
	ST342(41-60-13-1-5-4-14)				1	**1**	0		
CC109	ST345(26-4-2-2-9-2-5)		1		1	**2**	1	3	2
	ST109(26-4-2-2-9-1-5)	1				**1**	1		
CC107	ST107(34-35-37-1-5-6-36)			1		**1**	1	2	2
	ST1142(34-4-37-1-5-6-36)	1				**1**	1		
CC494	ST1422(3-3-2-5-29-1-4)				1	**1**	0	2	1
	ST494(3-3-2-5-4-1-4)			1		**1**	1		
CC172	ST172(1-16-2-6-18-4-4)				1	**1**	0	2	0
	N9(1-16-2-6[#]-18-4-4)				1	**1**	0		
CC639	ST639(3-3-2-2-11-57-4)			1		**1**	1	2	1
	N17(3-37-2-2-11-57-4)				1	**1**	0		
-	ST499(5-2-39-2-3-1-5)		29		4	**33**	29		
-	ST164(40-3-7-2-40-4-4)	1	13			**14**	14		
-	ST636(2-1-2-2-2-1-1)		10			**10**	10		
-	ST32(1-1-2-2-3-4-4)	1	2	3	2	**8**	6		
-	ST40(1-2-2-2-5-1-14)	1		1	4	**6**	2		
-	ST126(3-2-7-2-7-1-3)			1	4	**5**	1		
-	ST150(39-2-2-2-4-27-4)			2	3	**5**	2		
-	ST16(7-7-2-2-8-4-4)	3		1	1	**5**	4		
-	ST215(27-2-7-2-2-1-2)		3			**3**	3		
-	ST331(3-2-2-2-7-2-5)				3	**3**	0		
-	N10(1-2-2-1-11-2-4)				2	**2**	0		
-	ST108(35-1-11-7-9-25-2)	2				**2**	2		
-	ST1093(1-158-2-2-165-1-2)		2			**2**	2		
-	ST1547(13-3-7-3-7-1-16)				2	**2**	0		
-	ST162(3-2-2-2-2-4-8)	2				**2**	2		
IC3/-	ST229(3-3-51-2-28-1-3)		2			**2**	2		
-	ST267(12-37-2-2-3-2-14)				2	**2**	0		
-	ST338(8-5-5-26-13-1-2)			2		**2**	2		
-	ST647(3-3-6-2-51-1-29)				2	**2**	0		
-	N11(13-3[#]-7-26-7-1-29)		1			**1**	1		
-	N12(3-1[#]-7-1-9-1-4)				1	**1**	0		
-	N16(3-3-6-2-102-4-4)				1	**1**	0		
-	N19(5-1-101-2-5-1-5)				1	**1**	0		
-	N6(50-3[#]-6[#]-1-3-4-4)		1			**1**	1		
-	N7(13-4-5-3-6-1-29)		1			**1**	1		
-	ST111(3-3-2-2-4-8-12)	1				**1**	1		
-	ST1197(1-4-2-2-7-58-2)				1	**1**	0		
-	ST1336(1-3-40-2-7-1-1)			1		**1**	1		
-	ST1384(1-3-194-2-4-4-5)				1	**1**	0		
-	ST142(13-4-40-1-42-1-16)				1	**1**	0		
-	ST1472(3-1-5-26-13-2-3)	1				**1**	1		
-	ST152(8-1-5-3-6-2-3)				1	**1**	0		
-	ST1542(1-4-5-3-6-2-3)				1	**1**	0		
-	ST1545(3-1-7-5-3-1-4)				1	**1**	0		
-	ST1546(3-3-15-5-71-1-36)				1	**1**	0		
-	ST1554(3-3-2-79-3-4-4)			1		**1**	1		
-	ST1572(2-2-2-2-5-2-1)		1			**1**	1		
-	ST221(3-1-2-1-18-1-48)				1	**1**	0		
-	ST2247(25-3-7-2-5-4-5)				1	**1**	0		

Clonal Cluster	MLST(Pasteur)*	ISAba1 in Chromosome/ Plasmid				SUM	ISAba1(+)	CC SUM	CC ISAba1(+)
		+/+	+/-	-/+	-/-				
-	ST2252(5-4-2-1-3-1-5)		1			**1**	1		
-	ST2253(3-4-2-2-5-2-4)	1				**1**	1		
-	ST2266(8-1-56-3-7-1-4)				1	**1**	0		
-	ST2276(3-2-2-34-5-1-14)				1	**1**	0		
-	ST2532(3-1-5-3-3-1-2)				1	**1**	0		
-	ST274(40-2-2-2-9-1-36)				1	**1**	0		
-	ST282(3-3-2-6-52-2-14)				1	**1**	0		
-	ST285(1-52-2-2-9-4-2)				1	**1**	0		
-	ST294(40-3-2-2-4-35-4)				1	**1**	0		
-	ST309(12-1-2-2-9-1-5)				1	**1**	0		
-	ST350(3-3-2-2-5-2-5)				1	**1**	0		
-	ST400(3-3-55-2-66-1-5)				1	**1**	0		
-	ST412(1-52-2-2-67-4-5)		1			**1**	1		
-	ST477(26-2-6-2-5-1-5)				1	**1**	0		
-	ST479(3-3-11-2-44-4-8)				1	**1**	0		
-	ST49(3-3-6-2-3-1-5)	1				**1**	1		
-	ST54(12-3-18-2-17-4-5)			1		**1**	1		
-	ST57(1-3-17-5-3-1-14)				1	**1**	0		
-	ST578(97-3-13-1-4-4-14)				1	**1**	0		
-	ST638(100-3-14-1-7-1-4)		1			**1**	1		
-	ST648(1-3-11-5-111-1-14)				1	**1**	0		
-	ST649(27-3-2-2-5-58-5)	1				**1**	1		
-	ST690(3-3-2-1-7-2-14)				1	**1**	0		
-	ST738(3-3-105-6-4-2-5)				1	**1**	0		
-	ST756(12-1-7-1-7-2-29)				1	**1**	0		
-	ST866(3-2-2-30-3-74-3)			1		**1**	1		
	SUM	203	579	33	116	**931**	815		

* New STs were named with Arabic numerals beginning with the letter N. For the ones no distinct alleles were assigned in the seven loci, the closest alleles were selected and marked by "#".
